# Common recognition topology of mex transporters of *Pseudomonas aeruginosa* revealed by molecular modelling

**DOI:** 10.3389/fphar.2022.1021916

**Published:** 2022-11-11

**Authors:** Andrea Catte, Venkata K. Ramaswamy, Attilio Vittorio Vargiu, Giuliano Malloci, Andrea Bosin, Paolo Ruggerone

**Affiliations:** Department of Physics, University of Cagliari, Monserrato (CA), Italy

**Keywords:** RND efflux pumps, multidrug transporter, *Pseudomonas aeruginosa*, antibiotic resistance, molecular dynamics, molecular modeling

## Abstract

The secondary transporters of the resistance-nodulation-cell division (RND) superfamily mediate multidrug resistance in Gram-negative bacteria like *Pseudomonas aeruginosa*. Among these RND transporters, MexB, MexF, and MexY, with partly overlapping specificities, have been implicated in pathogenicity. Only the structure of the former has been resolved experimentally, which together with the lack of data about the functional dynamics of the full set of transporters, limited a systematic investigation of the molecular determinants defining their peculiar and shared features. In a previous work (Ramaswamy et al., Front. Microbiol., 2018, 9, 1144), we compared at an atomistic level the two main putative recognition sites (named access and deep binding pockets) of MexB and MexY. In this work, we expand the comparison by performing extended molecular dynamics (MD) simulations of these transporters and the pathologically relevant transporter MexF. We employed a more realistic model of the inner phospholipid membrane of *P. aeruginosa* and more accurate force-fields. To elucidate structure/dynamics-activity relationships we performed physico-chemical analyses and mapped the binding propensities of several organic probes on all transporters. Our data revealed the presence, also in MexF, of a few multifunctional sites at locations equivalent to the access and deep binding pockets detected in MexB. Furthermore, we report for the first time about the multidrug binding abilities of two out of five gates of the channels deputed to peripheral (early) recognition of substrates. Overall, our findings help to define a common “recognition topology” characterizing Mex transporters, which can be exploited to optimize transport and inhibition propensities of antimicrobial compounds.

## Introduction

*Pseudomonas aeruginosa* is the leading cause of hospital-acquired infections worldwide due to the emergence and spread of multidrug-resistant strains susceptible to very few antimicrobial agents (Fischbach and Walsh, 2009; Poole, 2011). *P. aeruginosa* resistance to multiple antibiotics arises from the synergy between its low permeable outer membrane and the action of multidrug efflux systems, among which the resistance-nodulation-cell division (RND) superfamily of secondary transporters plays a major role (Hancock, 1998; Li et al., 2015).

RND transporters responsible for the export of multiple drugs in Gram-negative bacteria belong to the substrate-based subfamily known as the hydrophobe/amphiphile efflux (HAE) family (Nikaido, 2018), which contribute to both intrinsic and acquired resistance (Poole et al., 1993; Poole, 2001, Poole, 2005; Poole and Srikumar, 2001; Dreier and Ruggerone, 2015). They function as tripartite efflux systems constituted by an inner membrane protein (IMP) recognizing the substrates, a periplasmic membrane fusion protein (MFP, aka PAP), involved in the assembly and/or substrate transfer, and an outer membrane channel protein (OMP) shuttling the substrates to the extracellular environment of the bacterial cell (Alav et al., 2021; Klenotic et al., 2021). The extrusion of substrates is powered by the proton motive force, which provides the energy required for the transport of different antibiotics and toxic compounds.

The first member of the RND family in *P. aeruginosa*, namely the MexAB-OprM transporter, was identified about 30 years ago (Poole et al., 1993). Since the first structural study of AcrB [the homologue of MexB and the main RND transporter in *Escherichia coli* and other bacteria—(Kobylka et al., 2020)] published in 2002 (Murakami et al., 2002), many experimental structures of individual components as well as the assemblies of MexAB-OprM and AcrAB-TolC have been reported (Sennhauser et al., 2009; Nakashima et al., 2013; Eicher et al., 2014; Tsutsumi et al., 2019; Glavier et al., 2020) (see for instance (Alav et al., 2021) and (Klenotic et al., 2021) for a full list of references).

**FIGURE 1:**
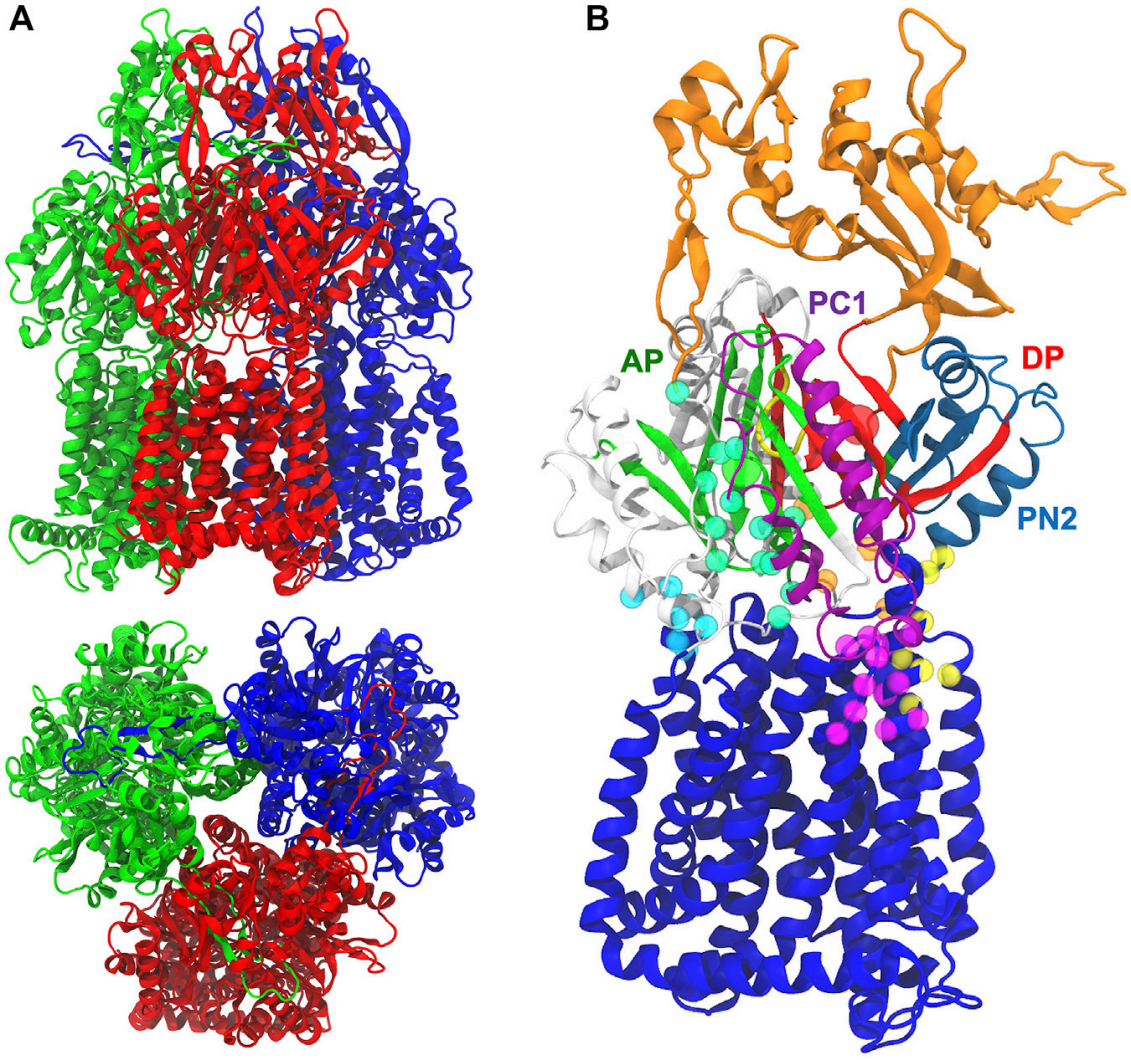
**(A)** Side and top views of MexB X-ray crystal structure (PDB ID 3W9I) Loose (L; red), Tight (T; blue), and Open (O; green) protomers are shown in different colors. **(B)** The pre-MD structure of the MexF Open protomer. The funnel and inner transmembrane domains are shown in orange and blue, respectively. AP and DP binding sites are in green and red, respectively, and their geometrical centers are represented by spheres. PC1 and PN2 subdomains are in purple and skyblue, respectively. The G-loop is in yellow. All other periplasmic residues are shown in white. CH1, CH2, CH3, CH4, and CH5 domains are highlighted with cyan, seagreen, orange, yellow, and magenta, respectively, spheres having as centers the position of alpha carbons.

MexB shows a characteristic jellyfish-like morphology constituted by an asymmetric trimer with each protomer comprising three domains (Figure 1A) (Ruggerone et al., 2013): 1) A transmembrane domain (TMD) of 12 α-helices inserted into the inner membrane (IM), where the chemical energy of protonated aspartates is converted into motion; 2) a pore (porter) domain (PD) located in the periplasm, where the recruitment and transport of substrates occur; and 3) a periplasmic funnel domain (FD), which connects the RND transporter to the OMP *via* the assembly of MFPs (Symmons et al., 2015; Wang et al., 2017; Tsutsumi et al., 2019; Glavier et al., 2020; Chen et al., 2022). The transport of substrates occurs through a “functional rotation mechanism” in which protomers cycle in a concerted (not necessarily synchronous) fashion across three asymmetric states: Loose (L or access) in which a substrate binds to a peripheral site termed access (or proximal) pocket (AP_L_); Tight (T or binding) in which the substrate binds to a deep (aka distal) pocket (DP_T_); and Open (O or extrusion) in which the substrate is released into the central funnel leading toward the extracellular space *via* different OMP proteins (Murakami et al., 2006; Seeger et al., 2006; Pos, 2009). The protein pockets AP_L_ and DP_T_ (Figure 1B) were previously identified in AcrB as the binding sites responsible for the recognition and selectivity of different types of substrates based on their molecular weight or chemical type (Nakashima et al., 2011; Kobayashi et al., 2014; Schuster et al., 2016; Iyer et al., 2022). These binding pockets are separated by a flexible G-rich switch loop, which facilitates the transport of high-molecular-mass compounds from the AP_L_ (Nakashima et al., 2011; Eicher et al., 2012; Cha et al., 2014). A stretch of phenylalanine residues (namely F136, F178, F610, F615, F617, and F628), also known as the hydrophobic trap (HP-trap) and located in the DP_T_, were found to interact with substrates and moreover to stabilize inhibitors binding to the periplasmic region of the transporter (Vargiu and Nikaido, 2012; Sjuts et al., 2016).

Importantly, the DP_T_ binding site was also observed experimentally in MexB (Nakashima et al., 2013), and both the AP_L_ and DP_T_ were involved in the binding of substrates to the AdeB transporter of *Acinetobacter baumannii* (Morgan et al., 2021; Ornik-Cha et al., 2021). Thus, both the AP_L_ and the DP_T_ seem to be shared binding spots across HAE-1 transporters that are deputed to the recognition of substrates and inhibitors (Nakashima et al., 2013; Sjuts et al., 2016).

Structural studies on AcrB also identified up to five channels leading from the surface of the protein to the AP_L_ and/or DP_T_: they are located at the membrane/periplasm interface (CH1), in the periplasm (CH2), at the interface between the periplasm and central cavity of the T protomer (CH3), within the interface between PN2 and PC1 domains (CH4) and at the membrane/periplasm interface adjacent to CH4 (hereafter CH5); Figure 1B). These channels appear to be crucial for the early recognition of substrates and for polyspecificity (Zwama et al., 2018; Alav et al., 2021): CH1 is likely mediating the transport of detergents and small drugs, such as β-lactams (Murakami et al., 2006; Sennhauser et al., 2007; Pos, 2009; Eicher et al., 2012); CH2 leading from the periplasm to the AP has been suggested to transport high molecular weight drugs such as macrolides (Seeger et al., 2006; Sennhauser et al., 2007, Sennhauser et al., 2009; Zwama et al., 2018); CH3 is possibly involved in sequestering planar aromatic cations, such as ethidium bromide or rhodamine 6G (Zwama et al., 2018); CH4 has been proposed to transport carboxylated drugs, such as fusidic acid and β-lactam antibiotics (Oswald et al., 2016). CH5, earlier named S1’, was also proposed by a recent *in silico* investigation as a putative binding site for fusidic acid and carboxylated β-lactams in AcrB (Tam et al., 2020), and later shown to catalyze allosteric binding of multiple molecules of fusidic acid to the transmembrane domain binding pocket (Tam et al., 2021). The substrate specificity of *P. Aeruginosa* MexB is very similar to that of AcrB from *E. coli*, as evidenced by its ability to transport macrolides such as erythromycin, β-lactams, chloramphenicol, ethidium bromide, fluoroquinolones, detergents, tetracyclines, etc. (Li et al., 1995). Previous experimental studies of MexB and its homologues (in particular, MexF and MexY transporters) were devoted to the identification of the amino acids (Middlemiss and Poole, 2004; Wehmeier et al., 2009) and domains responsible for substrate recognition (Tikhonova et al., 2002; Eda et al., 2003b), the substrates specificities of the different Mex efflux pumps (Masuda et al., 2000; Collu et al., 2012), and the structural basis of the binding of inhibitors to MexB and MexY (Nakashima et al., 2013). MexY, which is expected to be structurally similar to MexB and AcrB (Murata et al., 2002; Eda et al., 2003a), seems to transport aminoglycosides, such as gentamicin, tobramycin, amikacin, and isepamicin much more efficiently than MexB (Krahn et al., 2012; Lau et al., 2014; Dreier and Ruggerone, 2015). MexF, the third most relevant transporter in *P. aeruginosa*, is highly expressed in *nfxC* mutants (Llanes et al., 2011) to confer increased resistance to quinolones/fluoroquinolones, chloramphenicol, trimethoprim, and tetracycline (Vaccaro et al., 2008; Koch et al., 2013; Dreier and Ruggerone, 2015), as well as imipenem and some β-lactamase inhibitors (Kohler et al., 1997a; Li et al., 1998; Köhler et al., 1999). Systems overexpressing the MexEF-OprN efflux pump have been suggested to be among the first-step mutants induced by exposure to the aforementioned compounds, which may promote the acquisition of additional resistance mechanisms (Poole, 2011).

The detailed information provided by structural studies enabled, with the aid of computer simulations, to shed some light into the molecular mechanisms of substrate recognition and transport by, as well as inhibition of RND transporters (Schulz et al., 2010; Vargiu et al., 2011, Vargiu et al., 2014; Collu et al., 2012; Ramaswamy et al., 2016, Ramaswamy et al., 2017b, Ramaswamy et al., 2017a, Ramaswamy et al., 2018; López et al., 2017; Atzori et al., 2019; Tam et al., 2020). However, while all Gram-negative bacteria are endowed with several such pumps, only the structure of one protein per bacterium has been resolved so far [AcrB in *E. coli* and *Salmonella enterica* (Johnson et al., 2020), MexB in *P. aeruginosa* (Nakashima et al., 2013; Tsutsumi et al., 2019), AdeB in *Acinetobacter baumannii* (Morgan et al., 2021; Ornik-Cha et al., 2021)]. This limits the understanding of which general molecular determinants are responsible for the specificity of each transporter, as well as of the shared properties that enable recognition of the same class of substrates by different pumps. This is particularly true for the accurate mapping of substrate preferences (and overlap) between the different channels in the different transporters, as a characterization of these channels has been partially done only for AcrB.

We recently exploited MD simulations and fragment-based protein mapping to identify chemotype-specific and multi-functional sites (MFSs) (Imai et al., 2011) in the AP_L_ and DP_T_ of AcrB/AcrD from *E. coli* and of MexB/MexY from *P. aeruginosa* (Ramaswamy et al., 2017b, Ramaswamy et al., 2018). Focusing on the latter pathogen, in this study we present the first *in silico* comparative study of its main three transporters MexB, MexF, and MexY. State-of-the-art MD simulations were performed in multiple copies for each transporter, followed by analyses of their structural, dynamical, physico-chemical features, and the mapping of their putative hot-spots and MFSs. Importantly, this analysis covered not only the AP_L_ and the DP_T_, but also highlighted the preferential binding of different fragments to all the channel gates of each transporter.

## Materials and methods

### Homology modeling of MexF

Since there is no experimental structure of MexF, we built up an all-atom model of its asymmetric trimer structure by homology modeling using Modeler 9.13 (Sali and Blundell, 1993). The amino acid sequence of full length MexF transporter from *P. aeruginosa* PAO1 was retrieved from the UniProtKB database [(The Uniprot Consortium, 2015); https://www.uniprot.org] (UniProt ID Q9I0Y8), and subsequently searched for the best available template structures bearing homologous relationship to the query sequence using the NCBI-BLAST tool (Madden, 2013) against the Protein Data Bank (PDB, www.rcsb.org). The X-ray crystal structure of MexB at 2.7 Å resolution [PDB ID 3W9I (Nakashima et al., 2013)] was chosen as a template for the homology modeling of MexF. The protein sequences were optimally aligned by ClustalOmega (Sievers et al., 2011; Sievers and Higgins, 2014) and the results were visually inspected to ensure the absence of gaps in important secondary structure regions. Multiple sequence alignments were analyzed using EMBOSS Stretcher to calculate sequence identity and similarity (Rice et al., 2000). Modeler 9.13 was used to generate a total of 100 asymmetric models of MexF using an optimization method combining slow MD with a very thorough variable target function method through 300 iterations. This whole cycle was repeated twice unless the objective function (MOLPDF) was greater than 10. The resulting models were ranked using discrete optimized protein energy (DOPE) (Shen and Sali, 2006) score values, and the top 5 models (with the lowest DOPE score) were selected for individual structure quality checks. Each model was further subjected to loop refinement using Modeler, and to overall structure relaxation by energy minimizations using AMBER18 (Case et al., 2018). The most reliable model was then selected based on various geometric and stereochemical quality factors evaluated for backbone angles, side chains flips, rotamers, steric clashes etc. using PROCHECK (Laskowski et al., 1993), ERRAT (Colovos and Yeates, 1993), ProSA (Wiederstein and Sippl, 2007), Verify3D (Eisenberg et al., 1997) programs available in MolProbity (Williams et al., 2018) and Structure Analysis and Verification Server. We performed comparative structural evaluation by superimposition of the modeled MexF structures over the experimentally determined X-ray crystal structure of MexB used as a template. Likewise, the template structure was also evaluated with the same programs to serve as reference for the results obtained for the MexF models (data not shown). Visual inspections were performed with VMD1.9.3 (Humphrey et al., 1996) and PyMOL (Schrödinger, 2015).

### MD simulations of MexF, MexB, and MexY

All-atom MD simulations of MexF, MexB, and MexY were carried out at 310 K and 1 atm using the MD software package AMBER18 (Case et al., 2018). The X-ray crystal structure of MexB (PDB ID 3W9I) (Nakashima et al., 2013) and the homology models of MexF and MexY will be hereafter referred to as pre-MD. The most reliable homology model of MexF obtained as described above was used as the starting structure. The details of the validation of the pre-MD model of MexF are reported in the Supplementary Table S1. We considered the highest resolution X-ray crystal structure released by Nakashima et al. (2013) (PDB ID 3W9I) and the validated homology model published by Ramaswamy et al., in 2018 as initial models for MexB (Figure 1A) and MexY, respectively. The protonation states of aspartate, glutamate and histidine sidechains were assigned by comparing the pK_a_ values calculated by PROPKA3 (Olsson et al., 2011) with the physiological pH experienced by each amino acid and considering its exposure to the solvent. As previously reported by Eicher et al., in 2014, we considered that periplasmic (i.e., H318 and H749 of MexF) and cytoplasmic (i.e., H525 and H534 of MexB and MexF, respectively) side chains exchanged protons with aqueous solutions at pH values of 5.5 and 7.5, respectively, while transmembrane domain residues (i.e., D407, D410 and D406 of MexB, MexF, and MexY, respectively) experienced intermediate pH values between these lower and upper limits (Eicher et al., 2014). Moreover, the ionizable residues exhibiting buried sidechains were left unprotonated. The protonation states of specific amino acids in each protomer (Eicher et al., 2014) were adopted with E346 (E349 and E345) and D923 (N935 and D919) protonated in both Loose (L) and Tight (T) protomers while deprotonated in the Open (O) protomer of MexB (MexF and MexY). The residues D407 (D410, D406), D408 (D411, D407), D566 (Q575, E563) and H338 (E341, R337) were protonated only in the Open protomer of MexB (MexF, MexY). MexF N935, Q575, and E341 and MexY R337 residues were not protonated. The periplasmic residues H318 and H749 were protonated only in all protomers of MexF, as a result of a pK_a_s analysis performed on MexB and its homologues using PROPKA3 version 3.4.0 (Olsson et al., 2011). The same protonation employed by Ramaswamy et al. (2018) for MexY was used. Each protein was successively embedded in 1-palmitoyl-2-oleoyl-sn-glycero-3-phosphatidylethanolamine (POPE): 1-palmitoyl-2-oleoyl-sn-glycero-3-phosphatidylglycerol (POPG) (POPE:POPG = 4:3) lipid bilayer patches, solvated with explicit OPC (Optimal Point Charges) water molecules (Tian et al., 2020). The ratios of POPE and POPG molecules in lipid membranes containing MexB, MexF, and MexY were 380:285, 356:267, and 388:292, respectively. The total number of solvation waters for lipid membranes containing MexB, MexF, and MexY was 96,936, 99,309, and 98,527, respectively. The residual charge of the systems was neutralized by appropriate numbers of randomly placed K^+^ and Cl^−^ ions to reach a physiological ionic strength of 0.15 M KCl (Schulz et al., 2010, Schulz et al., 2011, Schulz et al., 2015; Vargiu et al., 2011). The total number of atoms of each Mex transporter embedded in a hydrated lipid bilayer was about 520,000.

The ions count was suitably adjusted to set a physiological ionic strength of 0.15 M KCl. The insertion of the protein into a POPE:POPG lipid membrane was performed using the Packmol-Memgen software (Schott-Verdugo and Gohlke, 2019). The POPE:POPG molar ratio of 4:3 was chosen to mimic the planktonic lipid composition of the inner membrane of *P. aeruginosa* (Benamara et al., 2014). The topology and the initial coordinate files were created using the LEaP module of AmberTools20 (Case et al., 2021). Periodic boundary conditions were used and the distance between the protein and the edge of the box was set to be at least 30 Å in each direction. Multi-step energy minimization with a combination of steepest descent and conjugate gradient methods was carried out using the *pmemd* program implemented in AMBER20 to relax internal constraints of the systems by gradually releasing positional restraints. Following this, the systems were heated from 0 to 310 K by a 1 ns heating (0–100 K) under constant volume (NVT) followed by 5 ns of constant pressure heating (NPT) (100–310 K) with the phosphorous heads of lipids restrained along the *z*-axis to allow membrane merging and to bring the atmospheric pressure of the system to 1 bar. Langevin thermostat (collision frequency of 1 ps^−1^) was used to maintain a constant temperature, and multiple short equilibration steps of 500 ps under anisotropic pressure scaling (Berendsen barostat) in NPT conditions were performed to equilibrate the box dimensions. A time step of 2 fs was used during all these runs, while post-equilibrium MD simulations were performed with a time step of 4 fs under constant volume conditions after hydrogen mass repartitioning (Hopkins et al., 2015). The particle-mesh Ewald (PME) algorithm was used to evaluate long-range electrostatic forces with a non-bonded cutoff of 9 Å. During the MD simulations, the length of all R-H bonds was constrained with SHAKE algorithm. Coordinates were saved every 100 ps With respect to our previous study (Ramaswamy et al., 2017b), we employed updated atomistic models and more physiologically relevant lipid bilayers with a chemical composition similar to that of the inner membrane of *P. aeruginosa* (Benamara et al., 2014). The ff19SB (Tian et al., 2020) version of the all-atom Amber force field was used to represent the protein systems while Lipid17 (Gould et al., 2018) [an improved version of Lipid14 (Dickson et al., 2014)] parameters were used for the POPE:POPG lipid bilayer. After equilibration, multi-copy μs-long MD simulations were performed for each system, namely five ∼1 μs-long production simulations for each transporter (for a total simulation time of ∼15 µs). Trajectory analysis was done using *cpptraj* module of AmberTools20 and VMD1.9.3, and graphs were plotted using the xmgrace tool.

### Electrostatic potential

After pre-processing MexB, MexF, and MexY structures to assign charges and atomic radii using the PDB2PQR 3.5.0 (Dolinsky et al., 2004), the electrostatic potential surface maps were calculated using APBS 1.5 (Baker et al., 2001). All electrostatic potential calculations were performed at 0.15 M physiological salt concentration, with a solvent probe of radius 1.4 Å, a solvent dielectric constant of 78.5, a biomolecular dielectric constant of 2.0, a temperature of 310.15 K, a minimum grid spacing of 0.5 Å and keeping the other Poisson–Boltzmann parameters at default.

### Hydration analysis

The radial distribution function (rdf) indicates the probability of finding water molecules at a certain distance from a region or residue of interest and is commonly used to analyze the solution structure revealed from either experimental or computer simulations data. The rdf analysis of water oxygen atoms was performed using cpptraj module of AMBER20, in which the rdf is computed from the histogram of the number of solvent particles found as a function of the distance R from an (ensemble of) atom(s), normalized by the expected number of solvent particles at that distance in bulk. Bin spacing of 0.1 and a maximum bin value of 4.0 was used in this case to calculate the rdf of all water oxygen atoms to each atom of AP_L_ and DP_T_ over the entire length of the simulation.

### Clustering of MD simulation trajectories

A cluster analysis of MD trajectories was performed using the average-linkage hierarchical agglomerative clustering method implemented in the *cpptraj* module of AMBER20 (Case et al., 2021). Such clustering helps to reduce the number of structures for analysis while retaining the large conformational space sampled during MD simulations. In this approach, we clustered in thirteen separate instances the trajectory based on root mean square deviation (RMSD) (cutoff set to 3 Å) of AP_L_, DP_T_, CH1, CH2, CH3, CH4, and CH5 in both L and T protomers. The amino acids constituting each binding pocket and channel of investigated Mex transporters are reported in Supplementary Tables S2, S3, respectively. For each protein, the representative structures from each of the top 5 clusters generated in each of the thirteen different cases considered (AP_L_, DP_T_, CH1, CH2, CH3, CH4, and CH5 in L and T) were used to perform the fragment-based mapping (see below). All non-protein molecules were stripped from the trajectory during post-processing to reduce additional memory usage and to speed up file processing.

### Fragment-based mapping analysis of MexF, MexB, and MexY binding sites

Fragment-based methods exploit small organic ligands characterized by different physicochemical properties (Supplementary Figure S1) to identify putative hotspots for drug binding on a protein (Ciulli et al., 2006; Kozakov et al., 2015; Radoux et al., 2016; Wakefield et al., 2020; Hadfield et al., 2022; Smilova et al., 2022). These methods can be used to refine the choice of compounds to be screened in more accurate drug design investigations at both experimental and computational levels. The FTMap server (Kozakov et al., 2015) employed in this work implements the FTSite algorithm to discover druggable binding sites on proteins and to identify specific fragments that could serve as scaffolds for drug design attempts. FTMap identifies the important hot spots based on the consensus clusters of 16 standard probes, which include molecules varying in size, shape, and polarity (Supplementary Figure S1). Such a diverse library of probes captures a range of interaction types that include hydrophilic, namely *via* hydrogen bond donor (HBD) and acceptor (HBA) molecules, hydrophobic (aliphatic), and aromatic interactions. The regions where clusters of different probes of the same or different types overlap together are marked as CSs and MFSs, respectively, and are ranked based on the number of their clusters. Clusters near a top-ranked one are merged together and the protein residues within this region become the top-ranked putative ligand binding site. We identify as MFS a cluster of organic ligands displaying all types of interactions within 5 Å of any residue of the binding site of interest (Imai et al., 2011). The same cutoff of 5 Å has also been used to estimate the number of various types of organic probes interacting with different channels of each transporter.

### Lipophilic index calculations

In pharmaceutical science, the logarithm of the partition coefficient (P) between octanol and water phases (log P_o/w_) is one of the most used descriptors to adequately model the distribution between lipophilic cell membranes (octanol) and hydrophilic blood plasma (water) (Leo et al., 1971; Sangster, 1997). The three-dimensional distribution of lipophilicity on a molecular surface can be described using the molecular lipophilicity potential (MLP), which represents the lipophilic contributions of all polar and hydrophobic fragments of a molecule (Broto et al., 1984; Ghose and Crippen, 1986; Gaillard et al., 1994; Testa et al., 1996). To characterize the lipophilicity of AP_L_ and DP_T_ binding pockets in MexB, MexF, and MexY, we calculated the Lipophilic Index (LI) of pre-MD and the ten most populated cluster structures using the MLP-Pocket sub-program implemented in the MLP Tools plugin (Oberhauser et al., 2014) of PyMOL version 2.4.1 (Schrödinger, 2015). The location of each binding site was defined by its Cartesian coordinates and center. The cutoff was 4.0 Å for each calculation.

### Estimation of pockets volume

The volume of AP_L_ and DP_T_ binding pockets was monitored during MD simulations using the two-probe sphere method of *rbcavity* subprogram of the rDock suite (Ruiz-Carmona et al., 2014). This analysis provided detailed information on the pocket volume and plasticity of the site. The binding site volume was identified by a fast grid-based cavity detection algorithm (Morley and Afshar, 2004) within a sphere of radius 13 Å, centered over the pockets, using large and small probe radii of 6.0 and 1.5 Å, respectively.

## Results

### Sequence alignments comparison

Overall, the aminoacid sequences are well conserved across the strains of MexB, MexF, and MexY deposited in the UniProtKB database (accessed April 2021; Supplementary Figure S2). MexF displays the smallest sequence identity with both MexB and MexY (Supplementary Table S4). When analyzing in more detail the sequence conservation at key sites, we noticed that both the AP and DP of MexF and MexY are less conserved than the whole proteins, sharing respectively a percentage of identical (similar) residues of 37 (55) and 33 (54) % and 35 (48) and 31 (53) % with the corresponding site on MexB (Supplementary Figure S3). The AP of MexB and both the AP and the DP of MexY exhibit a more predominant distribution of negatively charged residues, while the balance is almost neutral in MexF (Supplementary Table S5). The AP of MexY also contains the lowest number of hydrophobic residues, while the AP and DP of MexF contain more polar residues than MexB and MexY. The HP-trap, lined by 6 phenylalanines in MexB, conserves its hydrophobic character in both MexF and MexY, although only 3 and 2 phenylalanines line the site in these proteins (Supplementary Figure S3). A similar comparison for the CHs sites reveals that (Supplementary Figure S3): CH1 is well conserved between MexB and MexF, while MexY displays several differences with respect to MexF and, moreover, MexB; CH2 turns out to be the less conserved entrance gate, with each transporter featuring a very dissimilar amino acid composition; at CH3, MexF displays a large similarity with MexY, and to a minor extent with MexB; CH4 and CH5 are overall the most conserved sites.

### MD simulations of MexB, MexF, and MexY

All-atom MD simulations of MexB, MexF, and MexY embedded in a POPE: POPG lipid bilayer were performed using as starting conformation the corresponding pre-MD structure, respectively the X-ray crystal structure of MexB (PDB ID 3W9I) (Nakashima et al., 2013) and the homology models of MexF and MexY. The stability of both MexF and MexY models and their suitability for subsequent quantitative analyses were further validated by performing five independent μs-long MD simulations (which were performed also for MexB). The analysis of Cα-RMSDs of the whole protein and individual protomers of each RND transporter, using as a reference their initial structure (Supplementary Figure S4), highlighted that a plateau was reached within at most ∼0.4 μs in all MD simulations. The results of several analyses performed on these equilibrated trajectories are reported below.

### Electrostatic potential at the binding pockets

**FIGURE 2:**
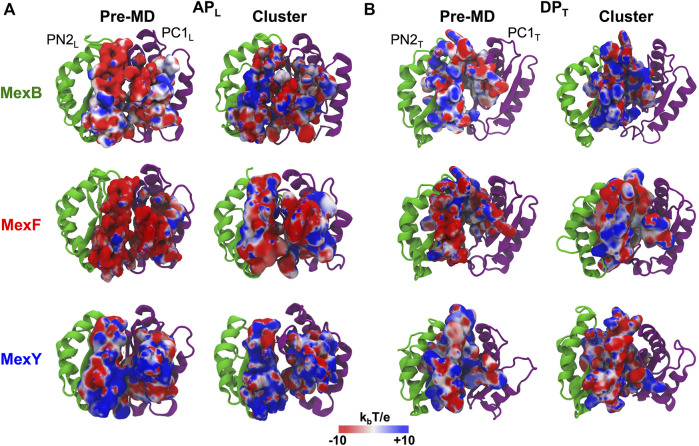
Electrostatic potential of **(A)** AP_L_ and **(B)** DP_T_ of MexB, MexF, and MexY. The electrostatic potential is plotted on the molecular surface representation of each binding pocket in the Pre-MD (left) and the most populated cluster (right) structures of MexB and its isoforms as seen from a periplasm. The color code is red to blue from negative (−10 k_*b*_T/e) to positive (+10 k_*b*_T/e) potentials, where k_*b*_ is the Boltzmann constant, T is the absolute temperature and e is the electron charge. PN2_L_, PC1_L_, PN2_T_, and PC1_T_ domains of each RND transporter are in green and purple cartoons, respectively.

MexF features an AP_L_ with the largest percentage of polar residues but the number of positively charged residues is very low compared to MexB and MexY (Supplementary Table S5), leading to a wider distribution of negatively charged patches of electrostatic potential in the pre-MD structure of the former transporter (Figure 2A). This difference in the distribution of charges at the AP_L_ persists, although less pronounced when comparing the most populated structure extracted from the cluster analysis of the MD simulations. In agreement with previous findings (Ramaswamy et al., 2018), we also observed a difference in the electrostatic potential surface at the AP_L_ between MexB and MexY, the latter exposing more positively charged patches (Figure 2A). The DP_T_ in pre-MD structures of MexF and MexB showed electrostatic potential surfaces more positive than those observed for the AP_L_, while MexY displayed an almost equal distribution of positive and negative charges (Figure 2B). Similarly to the AP_L_ results, the projection of the electrostatic potential onto the surface of the DP_T_ of most populated cluster structures resulted in a mainly positively charged environment in MexB and MexF as compared to MexY (Figure 2B), which is consistent with the relative percentages of positively vs. negatively charged residues in this site (Supplementary Table S5).

### Hydration of AP and DP binding sites

The hydration levels in binding sites of MexB and its homologs were evaluated by calculating the rdf of water oxygen atoms around AP_L_, DP_T_, and HP-trap_T_ residues (Supplementary Figure S5). As previously reported in (Ramaswamy et al., 2018), we observed a first solvation shell at around 2 Å for each binding site of MexB, MexF, and MexY. In particular, MexF has a slightly less hydrated AP_L_ than MexB and MexY. MexF, MexB, and MexY show comparable hydration profiles at the DP_T_. Interestingly, the HP-trap_T_ has similar hydration levels in the first shell of all transporters, while it is more hydrated in the second shell of MexF and MexY as compared to MexB. These results were confirmed by hydration analyses performed on MD simulations of the three RND transporters (Supplementary Figure S5).

### Fragment-based mapping of CSs and MFSs

In this work, we employed the FTMap server to probe AP and DP hot spots in different protomers of MexB, MexF, and MexY (Kozakov et al., 2015; Ramaswamy et al., 2017b, Ramaswamy et al., 2018). Among the putative binding sites identified with this methodology, the so-called consensus sites (CS; that is, regions on the protein where clusters of more than one probe of the same type do overlap, see Materials and Methods) and moreover the MFSs (that is, sites including all four kinds of probe functionalities: H-bond acceptor, H-bond donor, aliphatic, and aromatic) sites where probes of different kind cluster together) are particularly relevant for polyspecific transporters such as MexB and homologs (Kozakov et al., 2015; Ramaswamy et al., 2017b, Ramaswamy et al., 2018).

**FIGURE 3:**
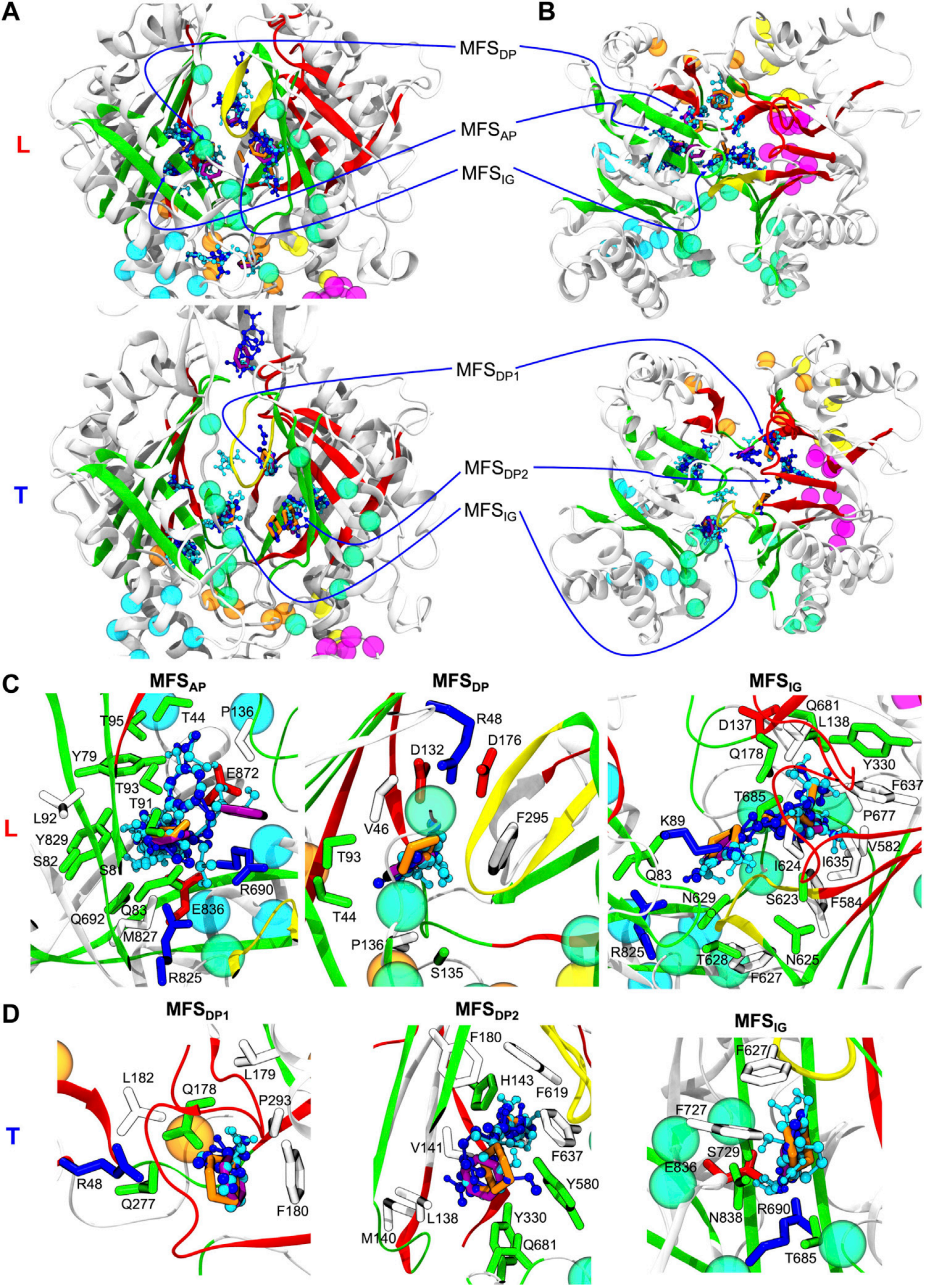
**(A)** Side and **(B)** top views of Loose (L) and Tight (T) protomers of the MexF pre-MD structure highlighting MFSs obtained with the FTMap server fragment-based mapping. Hydrogen bond donors, hydrogen bond acceptors, aliphatic, and aromatic organic probes are shown in celestial blue, blue, orange, and purple, respectively. The same color code of Figure 1 is applied to the protein and its channels. For the sake of clarity, only MFSs and CSs are shown. MFS_IG_ (IG: Interface/G-loop) is in proximity of the G-loop at the interface between AP and DP binding pockets. MFS_DP1_ and MFS_DP2_ denote the two different MFSs of the T protomer’s DP. **(C)** and **(D)** Zoomed views of MFSs located in L and T protomers, respectively, show sidechains of MexF residues interacting with each MFS, defined as those amino acids having at least one atom within 3.5 Å of any atom of the MFS small organic probes. Polar, negatively charged, positively charged, and hydrophobic sidechains are colored in green, red, blue, and white, respectively. For clarity, only MFSs of interest are shown in each panel, other adjacent MFSs and CSs are omitted.

**FIGURE 4:**
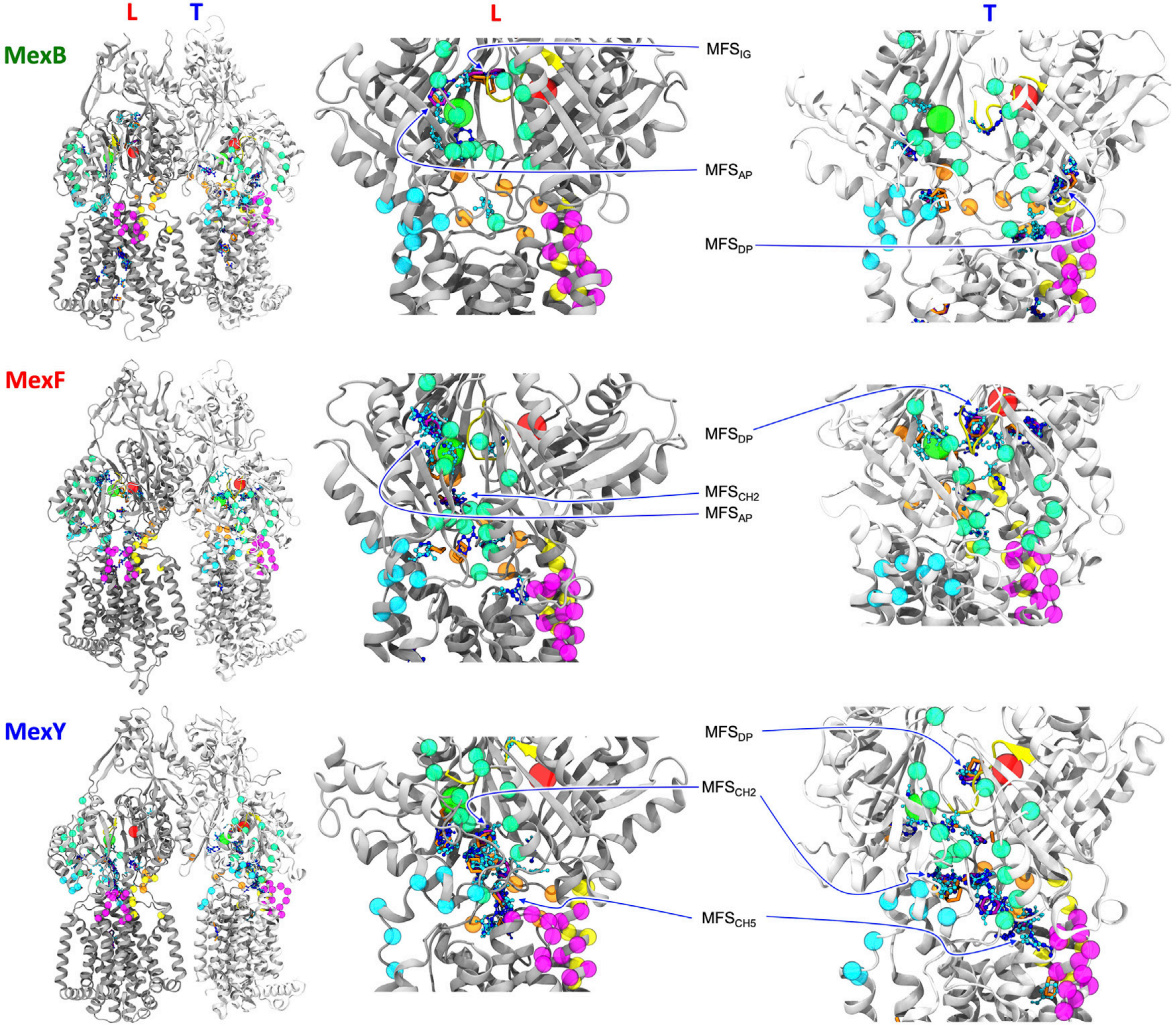
L and T protomers of MexB, MexF, and MexY best representative structures highlighting MFSs obtained with the FTMap server fragment-based mapping. Hydrogen bond donors, hydrogen bond acceptors, aliphatic and aromatic organic probes are shown with the same color code in Figure 3. For clarity, AP and DP binding sites are represented by green and red spheres, respectively, centered at the geometric center of each domain. The same color code of Figure 1 is applied to the protein binding pockets, switch loop and channels. All the other protein residues of L and T protomers are shown in gray and white, respectively. The definition of MFS_AP_, MFS_DP_ and MFS_IG_ is the same of Figure 3. MFS_CH2_ and MFS_CH5_ indicate MFSs in proximity of CH2 and CH5 channels, respectively. The sites not labeled as MFS are all CSs.

In the following, we will focus on the comparison between results obtained for MexF and the other two transporters. Two and one MFSs were detected respectively in the DP_T_ and in the AP_L_ of the pre-MD structure of MexF, increasing on average by 0.5 and 1 across the equilibrium MD trajectory (Table 1; Figures 3A,B). While these values are overall comparable with those obtained for MexB and MexY (Table 1; Supplementary Figures S6, S7, the number of MFS sites in the DP_T_ is the largest for MexF, which is in line with our findings on the average number of organic probes of any kind that can bind to this site in the different transporters (Supplementary Table S6). Both this site and the AP feature, in all transporters, a few hotspots for each type of organic probe: HBD, HBA, ALI, and ARO (Figure 4; Supplementary Table S6); a finding that is consistent with their polyspecificity. MexY features the largest (lowest) number of MFSs in the AP_L_ (DP_T_) among all the transporters, a result that is also consistent with the average number of hotspots found in these pockets (Table S6).

**TABLE 1:** MFSs identified in MexB, MexF, and MexY L and T protomers before (pre-MD) and along the MD simulation (average and maximum values are calculated over all replicas).

Structure		Number of MFSs							
		AP_L_	DP_T_	IG_L_	IG_T_	CH2_L_	CH2_T_	CH5_L_	CH5_T_
MexB	pre-MD	2	2	1	0	1	0	0	0
	MD	1.6^a^ (2)^b^	1.7 (3)	1.3 (3)	1.6 (2)	1.6 (3)	2.0 (3)	0.5 (2)	0.7 (1)
MexF	pre-MDact	1	2	1	1	1	1	0	0
	MD	1.8 (3)	2.6 (4)	1.6 (2)	1.4 (2)	1.5 (3)	1.5 (2)	0.2 (1)	0.8 (2)
MexY	pre-MD	1	0	0	0	0	3	1	0
	MD	2.0 (3)	1.0 (2)	1.9 (2)	1.5 (2)	2.0 (3)	2.1 (3)	1.2 (2)	1.0 (2)

^a^ Standard deviation of averages calculated over the best five structures of each RND, transporter produced by the clustering of MD, simulations and over the 5 replicas are all around 0.5.

^b^ Maximum number of MFSs, of L and T protomers are reported in parentheses.

Our data confirm the trend reported previously for MexB and MexY and show a more marked difference in the number of MFSs at DP_T_, whose average values also increased in both proteins with respect to ref. (Ramaswamy et al., 2018). These differences could be ascribed to the different MD simulations protocol employed (new water model and protein force field, see Materials and Methods) and/or the more complex lipid bilayer environment (i.e., the protein is inserted into a POPE:POPG (4:3) lipid membrane, in which neutral and, especially, negatively charged lipids can interact with the protein and affect its conformation in a way different from in a pure POPE lipid bilayer (Bruzzese et al., 2018; Liko et al., 2018). Moreover, our observation of MFSs in the transmembrane domain of each transporter (Supplementary Figure S8) could be affected by the chemical composition of the surrounding lipid environment, confirming the presence of cytoplasmic binding sites in MexB (Ohene-Agyei et al., 2012) and, also, in its homologs MexF and MexY.

For each Mex transporter, we also investigated the composition of the amino acids lining the MFSs (defined as the residues having at least one atom within 3.5 Å from any probe). In MexF, the MFSs within the AP_L_ (Figure 3C; Supplementary Figure S9) displayed more interactions with polar and charged residues than the MFSs in DP_T_ (Figure 3D; Supplementary Figure S9). Of note, the MFS_AP_ was in contact with several highly conserved residues between MexF (S81, S82, T91, T93, and E836) and MexB (S79, S80, T89, T91, and E825; see Supplementary Figure S6). In contrast, only one residue was shared between MexF and MexY at this site (E836—Q820 in MexY—see Supplementary Figure S7). During the MD simulations, despite the specific residues near the MFSs within the AP varied with respect to the initial conformation, the location of these MFSs did not change significantly (Supplementary Figure S9).

The MFS_DP_ in the T protomer of MexF is surrounded by several hydrophobic residues and, notably, by three aspartic acid residues (i.e., D132, D137, and D176; Supplementary Figure S9). This excess of negative charges, which is even larger than that observed in MexY and MexB, could be related to a more favorable interaction with positively charged drugs, such as aminoglycosides. Conserved residues D132, A134 in MexF and E129, A131 in MexY were interacting with MFSs in the DP of their corresponding proteins (Supplementary Figure S9).

We also monitored the occurrence of MFSs near all channel gates (Figure 1B). Among the five channels, we found that CH2 and CH5 showed a more significant population of MFSs (considering either the values from pre-MD or the averages from MD simulations in Table 1), pointing to the possibility of establishing relatively strong interactions with different drugs even at peripheral sites on these transporters. Consistently, these two entrance gates also were able to accommodate, on average, the largest number of different types of small organic probes (Supplementary Table S6; Supplementary Figure S10). Notably, in all transporters, the average number of MFSs at CH2 was nearly comparable with that found at AP_L_. CH5 displayed the highest average number of MFSs in MexY; no MFSs were found in the pre-MD structures of the other two transporters. As for CH2, when estimating the average number of organic probes at CH5_T_, we noticed the same trend as for the MFS (Supplementary Table S6). This is interesting as both MexY and MexB have been proposed to recognize β-lactam antibiotics (Alav et al., 2021), and CH5_T_ was proposed to be a putative entry gate for carboxylated drugs from this family (Tam et al., 2020). Conserved residues L683 and T685 in MexF (L669 and S671 in MexY) were found in proximity to MFS_CH2_. Regarding CH5, we observed a non-vanishing number of MFSs only in the L and T protomers of MexY (Table 1 and Figure 4; Supplementary Figure S9). Residues E564, P665, L666, and G99 were found in the proximity of the MFSs in both protomers. CH3 and CH4 also feature some interaction with all probes, although to a reduced amount as compared to CH2 and CH5 (Supplementary Table S6). Finally, CH1 seems virtually unable to bind any probe.

### Lipophilic indexes of AP_L_ and DP_T_ binding sites

The AP_L_ of MexF displayed the largest LI value in the pre-MD structures (Supplementary Table S7). Concerning MexB and MexY, despite different absolute values, the same trend reported in (Ramaswamy et al., 2018) is confirmed by our analyses. As in that work, the conformational changes underwent by the transporters during MD simulations have a drastic effect on the values of the LI. While the LI trend for MexB and MexY remains the same despite a large increase in both values, a significant reduction occurred in the AP_L_ of MexF (Supplementary Figure S11). This is likely to be associated with an increase and a decrease, along the MD simulations, of the AP_L_ volume respectively in MexB/Y and MexF (Supplementary Table S8 and Supplementary Figure S12), which together with the chemical composition of the site influence the evaluation of the LI (Ramaswamy et al., 2017b). As far as regards the DP_T_, in the pre-MD structures, the largest LI value was again found for MexF, followed by MexB and MexY, featuring a value almost half of that in the other transporters (Supplementary Table S7). For this pocket too, a remarkable smoothing of the differences among the transporters occurred during the MD simulation (although MexF still displayed the largest LI value). Indeed, both MexY and MexB exhibited a larger decrease in the DP_T_ volume as compared to MexF (respectively ∼41% and ∼27% vs. ∼13%) as compared to their pre-MD structures (Supplementary Table S8; Supplementary Figure S12).

## Discussion and perspectives

The overexpression of MexEF-OprN in *P. aeruginosa* (Köhler et al., 1997b, Köhler et al., 1999) is associated with increased resistance to fluoroquinolones, tetracyclines, and chloramphenicol in cystic fibrosis patients (Linares et al., 2005), and is found in various hospital strains together with MexAB-OprM and MexXY-OprM (Wolter et al., 2004; Morita et al., 2015). The study of these RND transporters at a molecular level could aid the development of drugs and inhibitors able to increase the susceptibility of this efflux pumps and, at the same time, to reduce their resistance to various classes of antibiotics.

Prompted by this consideration and with the goal of getting insights into the molecular determinants accounting for the different specificities of these three Mex transporters, we performed a systematic comparison of the physico-chemical, structural, dynamical, and thermodynamical features of the AP_L_ and DP_T_ binding sites of MexB, MexY, and (for the first time) MexF. The number, strength, and spatial distribution of MFS at these sites determines the level of promiscuity of their interactions (Ciulli et al., 2006). As evidenced by Ramaswamy et al., in 2018, the MFSs in the DP_T_ of MexB are placed exactly at the binding positions observed for several substrates like minocycline (Murakami et al., 2006; Nakashima et al., 2011; Eicher et al., 2012), doxorubicin (Murakami et al., 2006; Eicher et al., 2012), and inhibitors like D13-9001 (Nakashima et al., 2013) and MBX (Sjuts et al., 2016). Furthermore, we focused for the first time on comparatively addressing the role of the different gates of the channels leading to the AP_L_ and the DP_T_ binding pockets (Alav et al., 2021). The importance of multiple gates on the surface of RND transporters for polyspecific transport has been highlighted by numerous investigations. For instance, in the AcrB protein from *E. coli* up to four channels have been implicated in the recognition and transport of different classes of substrates, including detergents and β-lactams (mediated by CH1 and CH4) (Murakami et al., 2006; Sennhauser et al., 2007; Pos, 2009; Eicher et al., 2012), macrolides (mediated by CH2) (Seeger et al., 2006; Sennhauser et al., 2007, Sennhauser et al., 2009; Zwama et al., 2018), ethidium bromide and rhodamine 6G (mediated by CH3) (Zwama et al., 2018), and fusidic acid and carboxylated drugs (mediated by CH4 and CH5) (Tam et al., 2020).

We performed state-of-the-art MD simulations of each transporter embedded in hydrated membranes composed of phospholipid mixtures resembling the true *P. aeruginosa* inner membrane (Benamara et al., 2014). These were followed by analyses of hydration, electrostatics, volumetric profiles, as well as a fragment-based mapping of each Mex protein to monitor the presence of possible MFSs using small organic probes featuring different physicochemical properties (Supplementary Figure S1) (Imai et al., 2011; Ramaswamy et al., 2018). Although CH4 and CH5 gates are overall the most conserved among all investigated Mex transporters, CH4 displays identity percentages between MexB and MexF, MexF and MexY, and MexB and MexY lower than those observed for CH5.

The electrostatic features of the AP_L_ and DP_T_ are overall in agreement with the known specificity for charged substrates transported by these proteins. The greater negative charge patch on the surface of DP_T_ in MexY is functional to attract positively charged aminoglycosides and disfavor negatively charged molecules; however, the presence of scattered positive charges may feebly favor the binding of β-lactams (especially zwitterionic). Likewise, MexF and especially MexB with a more positive electrostatic potential surface in the DP_T_ tend to attract negatively charged as well as zwitterionic β-lactams and extrude them with greater efficiency along with weakly acidic quinolones, such as cinoxacin and nalidixic acid. In comparison, cationic antibiotics such as oleandomycin, erythromycin, and puromycin are pumped out with low efficiency (Tikhonova et al., 2002). The observed changes in electrostatic potential surfaces of AP_L_ and DP_T_ among pre-MD and most populated cluster structures are related to (expected) conformational changes taking place in the transporters during the MD simulations (Simsir et al., 2021).

The analysis of the fragment-based mapping of MexF highlighted the presence of a few MFSs in locations equivalent to the AP_L_ and the DP_T_ of MexB and MexY (Ramaswamy et al., 2018), suggesting that these pockets mediate polyspecific recognition also in this member of the Mex family of transporters. The predominance of MFSs and moreover HBD and HBA probes found in the DP_T_ of MexF (Table 1; Supplementary Table S6), together with the balance between positively and negatively charged residues (Supplementary Table S5) and its higher LI compared to MexB and MexY (Supplementary Table S7), might be key for the recognition of amphipathic compounds such as fluoroquinolones, tetracycline, and small hydrophilic compounds such as chloramphenicol (Fukuda et al., 1995; Kohler et al., 1997a; Aires et al., 2002; Fetar et al., 2011). Recognition of low-molecular-mass compounds seems to be mainly mediated by the DP also for this transporter, as already suggested for other members. The AP_L_ features instead a comparable number of MFSs among all transporters, although the LI remains the highest for MexF when calculated on the pre-MD structures. This, together with the lower percentage of charged residues lining the AP in MexF compared to MexB and MexY (Supplementary Figure S3; Supplementary Table S5), might disfavor effective interactions with substrates rich in HBD and HBA groups (such as aminoglycosides and carbapenems). These differences in LI values could also be interpreted considering the relative percentages of polar and hydrophobic amino acids lining the MFSs. For instance, compared to MexF and MexB, in MexY the MFS_AP_ within the L protomer was surrounded by a large number of negatively charged residues (i.e., K79, D615, and D811), which could rationalize the efficient recognition of positively charged substrates by the latter transporter (Supplementary Figures S5, S7).

Additionally, a relevant number of MFSs at the interface (switch- or G-loop) between AP_L_ and DP_T_ of each transporter was detected (Table 1), confirming the general belief that this region is an intermediate step in the transport of substrates from the AP_L_ to the DP_T_ sites. Furthermore, we have observed that two channels among all, namely CH2 and CH5, are endowed with MFS in all the transporters (the latter mostly in MexY), suggesting their possible involvement in the early recognition of several small organic probes (and thus substrates, such as antibiotics, functionalized with these chemical groups). This is relevant also from a pharmaceutical perspective, as it points to the possibility of targeting these sites in drug design efforts (Collu et al., 2012; Kumar Roy and Patra, 2020)—for instance to inhibit the translocation of substrates from the periplasm to the AP_L_ and DP_T_ binding pockets—and protein engineering (Mao et al., 2002; Middlemiss and Poole, 2004; Krishnamoorthy et al., 2008; Ohene-Agyei et al., 2012; Wang et al., 2015; Kumar Roy and Patra, 2020; Marshall and Bavro, 2020; Zwama and Nishino, 2021; Cacciotto et al., 2022).

Overall, our results suggest that, despite the different specificities of the Mex transporters, recognition and transport in this family are mediated by a shared “topology” which accounts for redundancy but allows subtle sequence alterations at specific site to confer peculiar binding abilities to each member.

In perspective, we intend to expand this analysis to MexD, which is another important member of the Mex RND transporters family lacking detailed structural information (Gotoh et al., 1999), as well as to the transporters AcrB and AcrD from *E. coli*, in particular, to understand at the molecular level of detail how entry gates mediate polyspecific transport (Husain and Nikaido, 2010; Husain et al., 2011; Nakashima et al., 2011; Eicher et al., 2012; Oswald et al., 2016; Schuster et al., 2016; Zwama et al., 2018; Tam et al., 2020; Zwama and Nishino, 2021).

## Data Availability

The data presented in the study are deposited in the Zenodo repository, with associated DOI: 10.5281/zenodo.7274367.

## Glossary

AP: Access (aka Proximal) Pocket
CS: Consensus Site
DOPE: Discrete Optimized Protein Energy
DP: Deep (aka Distal) Pocket
FD: Funnel Domain
HAE: Hydrophobe/Amphiphile Efflux
HBA: Hydrogen Bond Acceptor
HBD: Hydrogen Bond Donor
HP-trap: Hydrophobic Trap
IG: Interface/G-loop
IM: Inner Membrane
IMP: Inner Membrane Protein
LI: Lipophilic Index
MD: Molecular Dynamics
MFP: Membrane Fusion Protein
MFS: Multi-Functional Site
MLP: Molecular Lipophilicity Potential
OMP: Outer Membrane Protein
OPC: Optimal Point Charges
P: Partition coefficient
PAP: Periplasmic Adaptor Protein
PD: Porter (aka Pore) Domain
PDB: Protein Data Bank
PME: Particle-Mesh Ewald
POPE: 1-palmitoyl-2-oleoyl-sn-glycero-3-phosphatidylethanolamine
POPG: 1-palmitoyl-2-oleoyl-sn-glycero-3-phosphatidylglycerol
rdf: Radial Distribution Function
RMSD: Root Mean Square Deviation
RND: Resistance-Nodulation-cell Division
