# Supplementary material for: Common recognition topology of mex transporters of Pseudomonas aeruginosa revealed by molecular modelling
Source: Front Pharmacol. 2022 Nov 11;13:1021916. doi: 10.3389/fphar.2022.1021916 (PMC9691783; doi:10.3389/fphar.2022.1021916)
Supplement: Supplementary file 1 [file DataSheet1.PDF]

# Supplementary Material

## Common recognition topology of Mex transporters of Pseudomonas aeruginosa revealed by molecular modelling

Andrea Catte,[1] Venkata K. Ramaswamy,[1] Attilio Vittorio Vargiu,[1,*] Giuliano Malloci,[1] Andrea Bosin,[1] and Paolo Ruggerone[1]

1. Department of Physics, University of Cagliari, Monserrato (Cagliari), Italy

E-mail: vargiu@dsf.unica.it

**Table of Contents**

**Figure S1.** List of small organic probes/ligands used for the fragment-based mapping by the FTMap server.

**Figure S2.** Multiple sequence alignment of MexB, MexF and MexY RND transporters.

**Figure S3.** Multiple sequence alignment, sequence identity and similarity of AP, DP, HP-trap, and CH1-CH5 domains of MexB, MexF and MexY RND transporters.

**Figure S4.** RMSDs of alpha carbons of the whole protein and individual protomers of MexB, MexF and MexY.

**Figure S5.** RDFs of water around $AP_L$, $DP_T$ and $HP_T$ residues of MexB, MexF and MexY calculated for all performed MD simulations of RND transporters.

**Figure S6.** MexB pre-MD structure highlighting CSs and MFSs obtained with the FTMap server fragment-based mapping.

**Figure S7.** MexY pre-MD structure highlighting CSs and MFSs obtained with the FTMap server fragment-based mapping.

**Figure S8.** Loose and Tight protomers of MexB, MexF and MexY best representative structures highlighting MFSs obtained with the FTMap server fragment-based mapping.

**Figure S9.** Zoomed views of MFSs located in L and T protomers of MexB, MexF and MexY showing amino acids interacting with each MFS.

**Figure S10.** Distributions of different types of organic probes bound to channel residues of MexF L, T and O protomers.

**Figure S11.** Distribution of lipophilic indexes of $AP_L$ and $DP_T$ of MexB, MexF and MexY over the cluster representatives extracted from replicas of MD simulations of Mex RND transporters.

**Figure S12.** Volumes of $AP_L$ and $DP_T$ binding pockets of MexB, MexF and MexY best structures extracted from the clustering analysis of MD simulations.

**Table S1.** Validation of the MexF homology model.

**Table S2.** Amino acid residues of AP, DP and HP-trap regions of MexB and its isoforms.

**Table S3.** Amino acid residues of CH1, CH2, CH3, CH4 and CH5 channels of MexB and its isoforms.

**Table S4.** Sequence identity and similarity between RND transporters of *E. coli* and *P. aeruginosa*.

**Table S5.** Chemical composition of AP and DP of MexB and its isoforms.

**Table S6.** Number of different types of small organic probes identified in proximity of different binding domains of L, T and O protomers of MexB, MexF and MexY during MD.

**Table S7.** Lipophilic Indexes of $AP_L$ and $DP_T$ of MexB, MexF and MexY.

**Table S8.** Volumes of $AP_L$ and $DP_T$ of MexB, MexF and MexY.

**Figure S1**

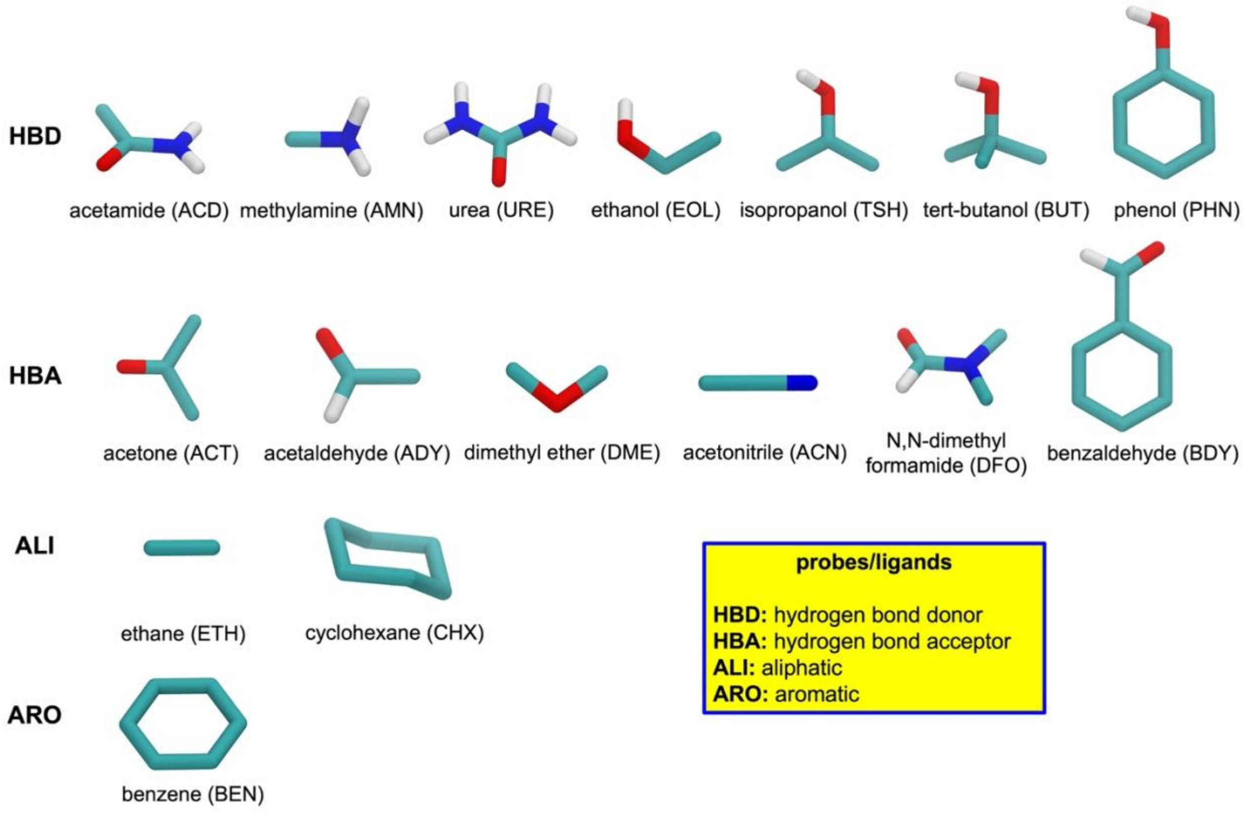


**Figure S1** List of small organic probes/ligands used for the fragment-based mapping by the FTMap server.

**Figure S2**

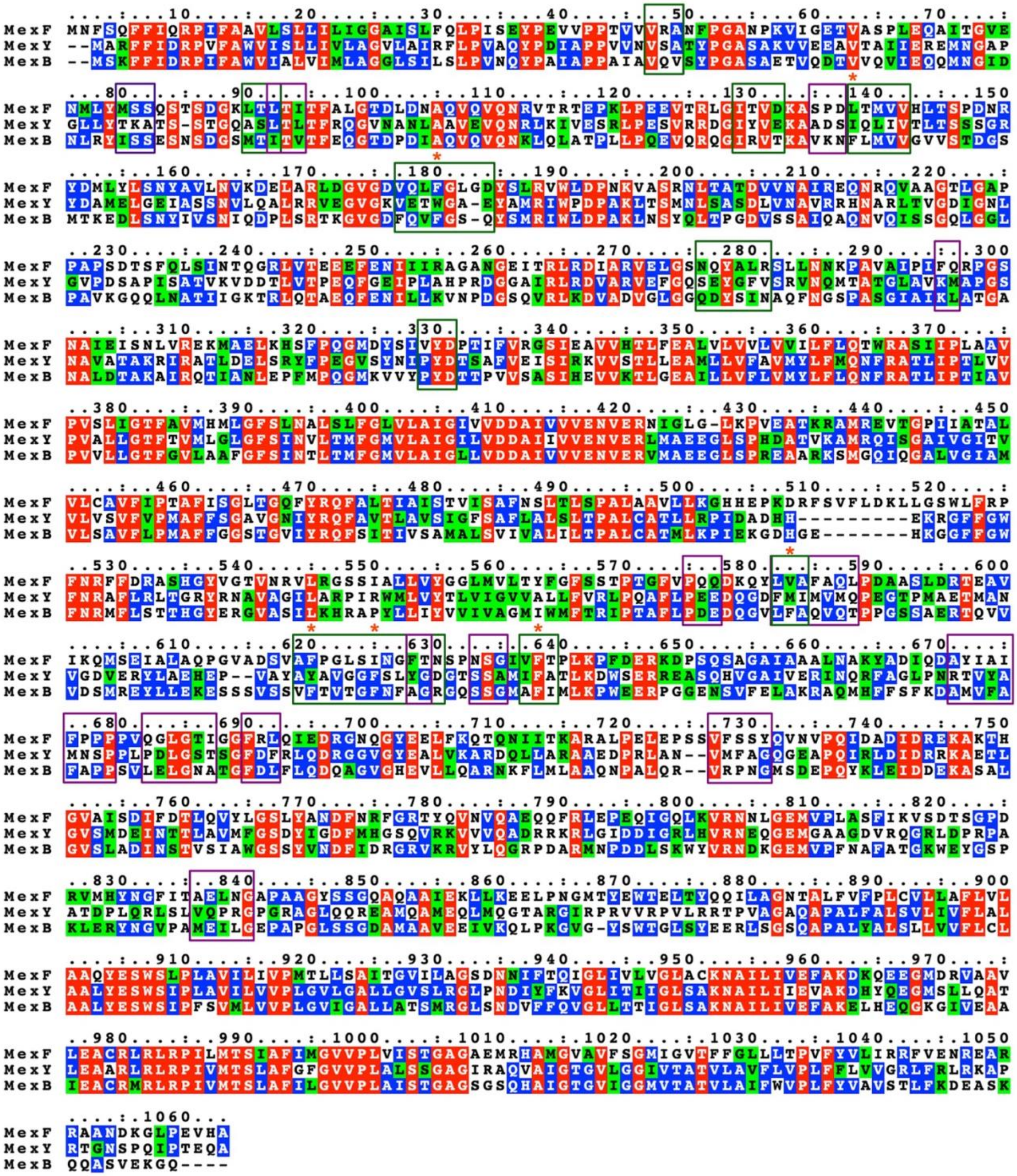

**Figure S2** Multiple sequence alignment of MexB, MexF, and MexY RND transporters. Conserved, identical, and similar residues are highlighted with red, blue, and green filled boxes, respectively. AP and DP residues are marked with purple and green boxes. Hydrophobic trap residues are highlighted by orange stars.

Figure S3

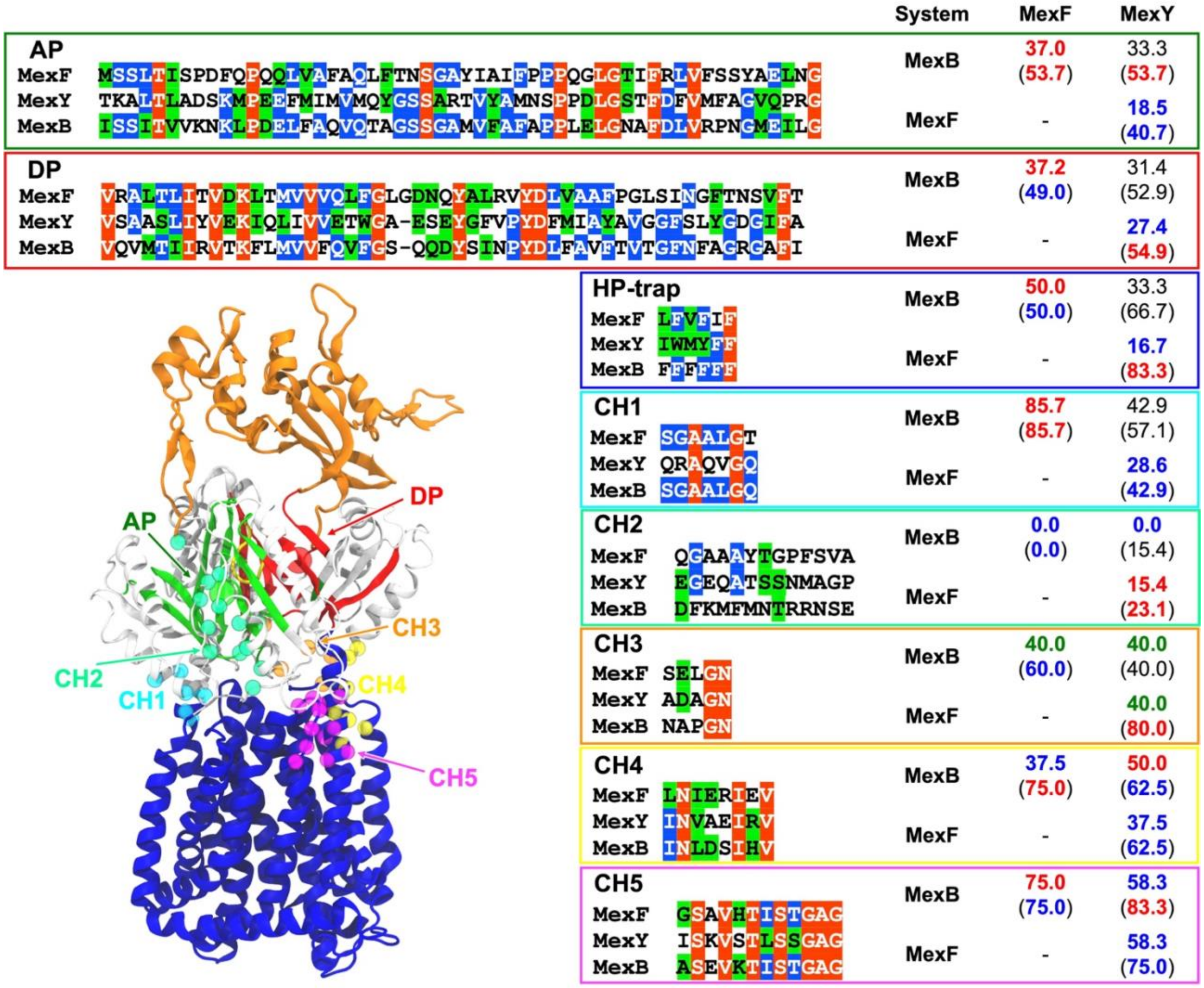


**Figure S3** Multiple sequence alignments of AP, DP, HP-trap, CH1, CH2, CH3, CH4, CH5 domains of MexB, MexF and MexY RND transporters. The color code for conserved, identical, and similar residues is the same of Figure S2. Sequence identities (similarities) are reported as percentages in the table. The largest, smallest, and identical sequence identities (similarities) are highlighted in bold red, blue and green characters, respectively. The left side panel shows the different domains of the MexF RND transporter.

**Figure S4**

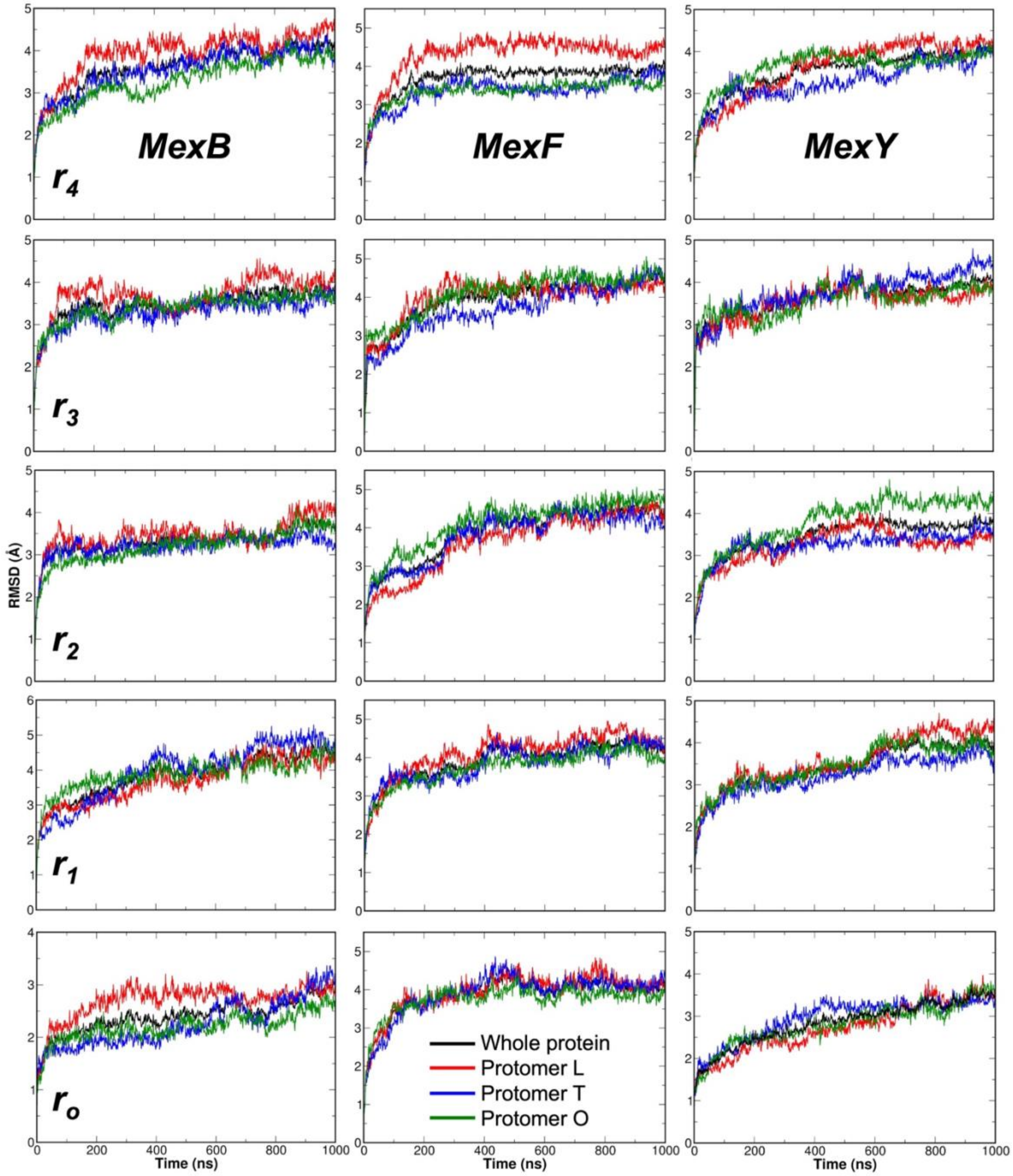


**Figure S4** $C_\alpha$-RMSDs calculated for the whole protein and individual protomers of MexB, MexF, and MexY RND transporters across the five MD simulations ($r_0$ to $r_4$) performed for each system.

**Figure S5**

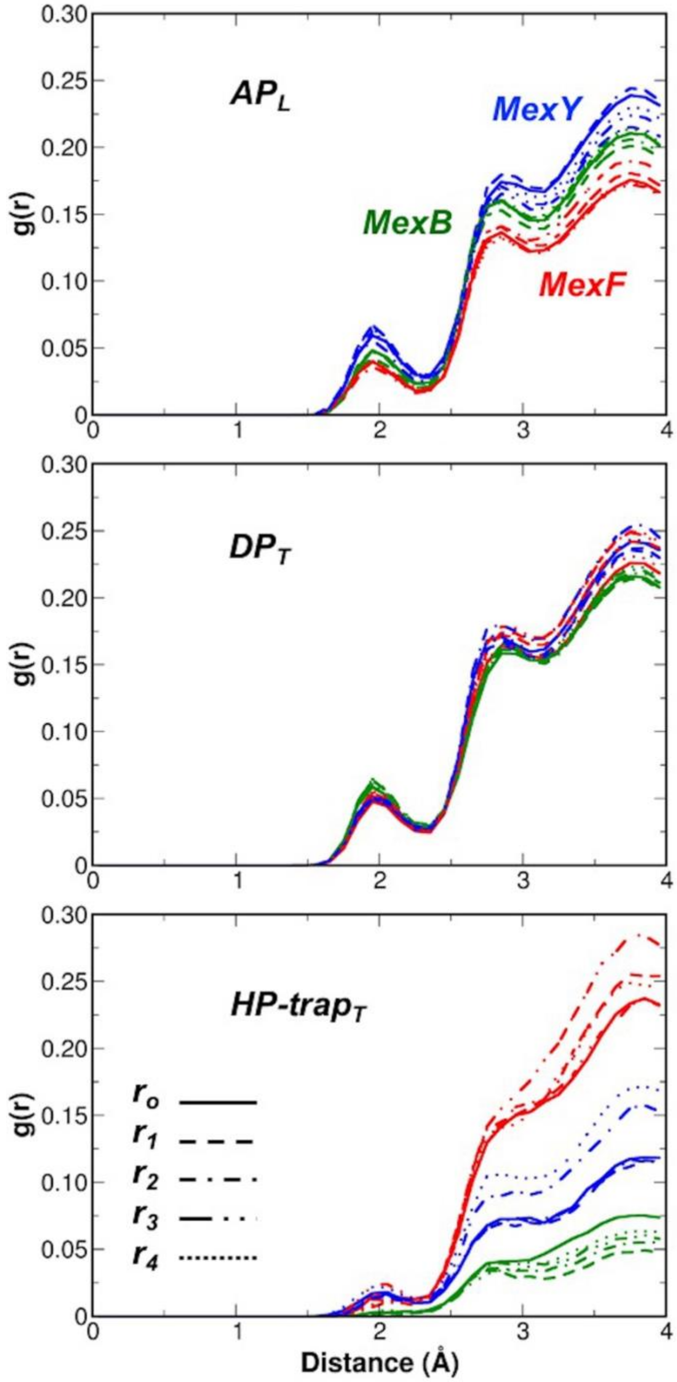


**Figure S5** Comparison of RDF profiles of water oxygen atoms around (top) $AP_L$, (middle) $DP_T$ and (bottom) $HP_T$ residues of MexB (green), MexF (red) and MexY (blue) in all performed MD simulations.

**Figure S6**

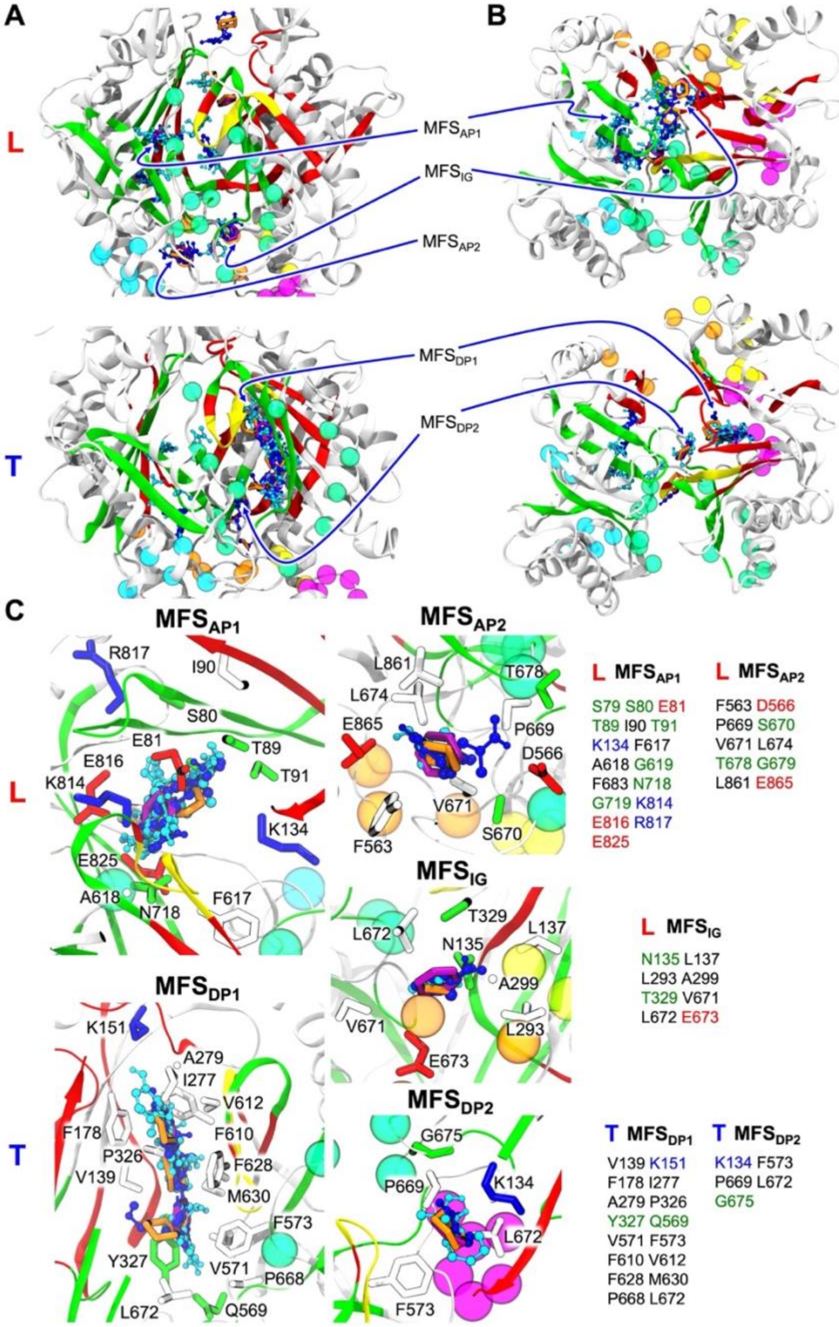


**Figure S6** (A) Side and (B) top views of Loose (L) and Tight (T) protomers of the MexB pre-MD structure highlighting MFSs obtained with the FTMap server fragment-based mapping. Hydrogen bond donors, hydrogen bond acceptors, aliphatic and aromatic organic probes are shown in celestial blue, blue, orange, and purple, respectively. The same color code of Figures 1, 3 and 4 is applied to the protein, its binding sites and channels. The sites not labeled as MFS are all CSs. $MFS_{AP1}$, $MFS_{AP2}$, $MFS_{DP1}$ and $MFS_{DP2}$ denote the different MFSs of $AP_L$ and $DP_T$, respectively. $MFS_{IG}$ is located in proximity of the G-loop at the interface between AP and DP binding pockets. (C) Zoomed views of MFSs located in L and T protomers, respectively, show sidechains of MexB residues (also reported as a list) within 3.5 Å of any atom of the MFS small organic probes as defined in Figure 3. Polar, negatively charged, positively charged, and hydrophobic sidechains are colored in green, red, blue, and white, respectively. For clarity, only MFSs of interest are shown in each panel, other adjacent MFSs and CSs are omitted.

**Figure S7**

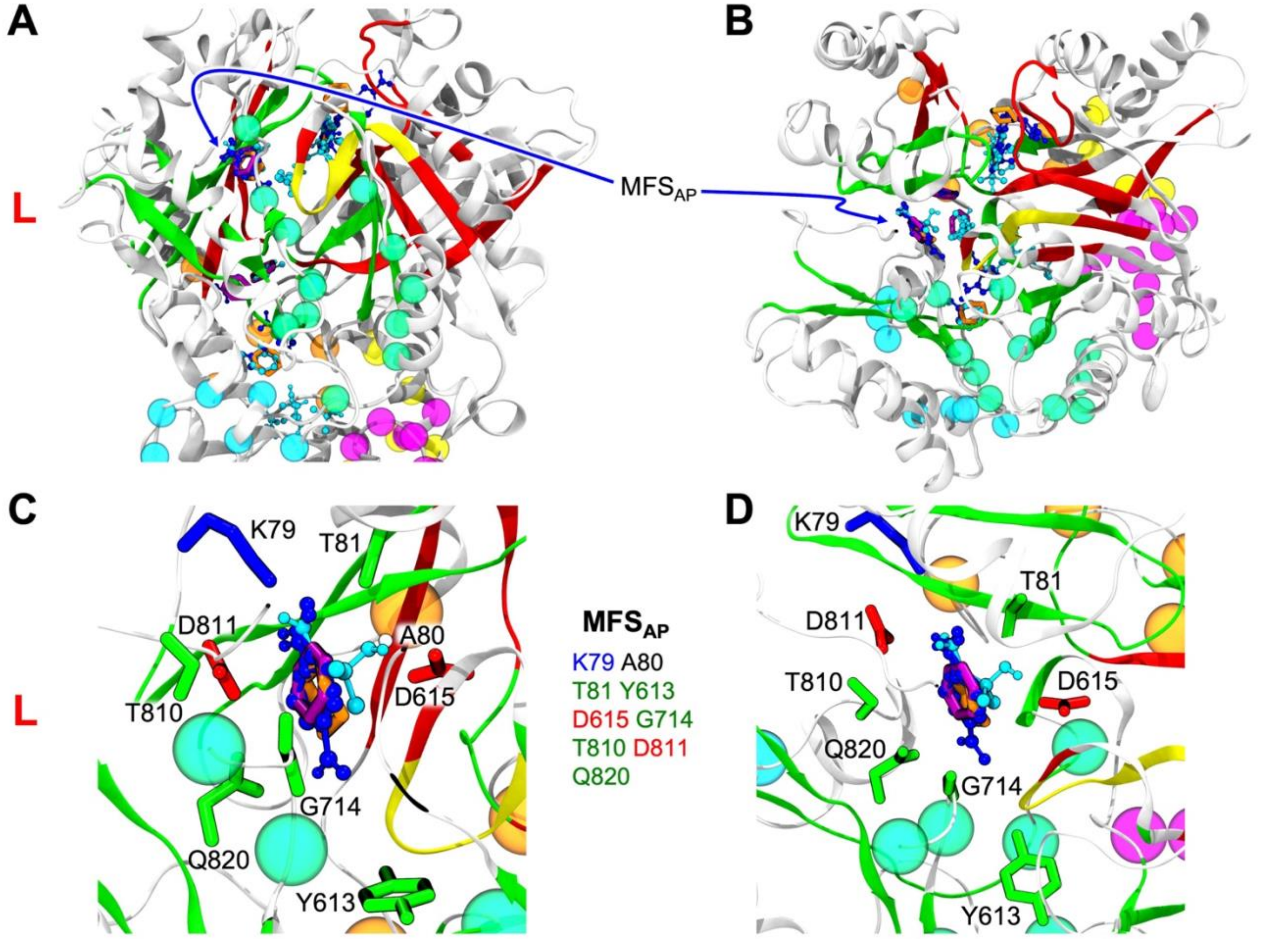


**Figure S7** (A) Side and (B) top views of Loose (L) protomer of the MexY pre-MD structure highlighting MFSs obtained with the FTMap server fragment-based mapping. (C) Side and (D) top zoomed views of the MFS$_{AP}$ located in the L protomer show sidechains of MexY residues (also reported as a list) within 3.5 Å of any atom of the MFS small organic probes as defined in Figure 3. See the caption of Figure S6 for details.

**Figure S8**

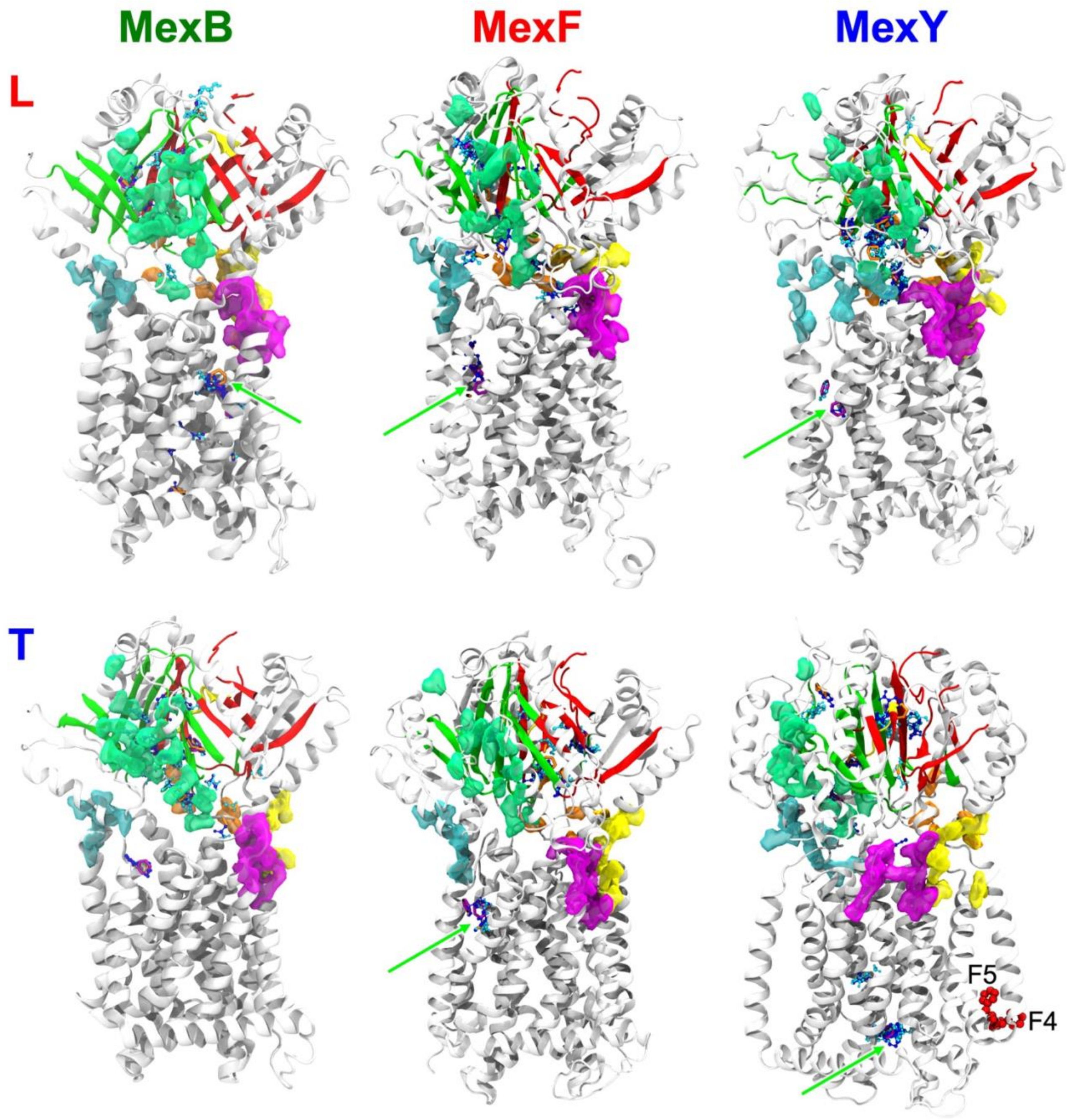


**Figure S8** (L) Loose and (T) Tight protomers of MexB, MexF and MexY best representative structures highlighting MFSs obtained with the FTMap server fragment-based mapping. Hydrogen bond donors, hydrogen bond acceptors, aliphatic and aromatic organic probes are shown with the same color code of Figure 3. The same color code of Figures 1, 3 and 4 is applied to the protein binding pockets, switch loop and channels. All the other protein residues are shown in white. MFSs located in the transmembrane domain ($MFS_{TMD}$) are indicated by green arrows. Note: MexF $MFS_{TMD}$ of L and T protomers are in proximity of the CH1 channel, while MexB $MFS_{TMD}$ of the L protomer is closer to the CH5 domain. MexY phenyalanine residues F4 and F5, which are highly conserved among Mex RND transporters (Figure S2) and previously reported by Ohene-Agyei *et al.* in 2012 to be relevant for the efflux of compounds acting within the cell, are shown as red spheres.

**Figure S9**

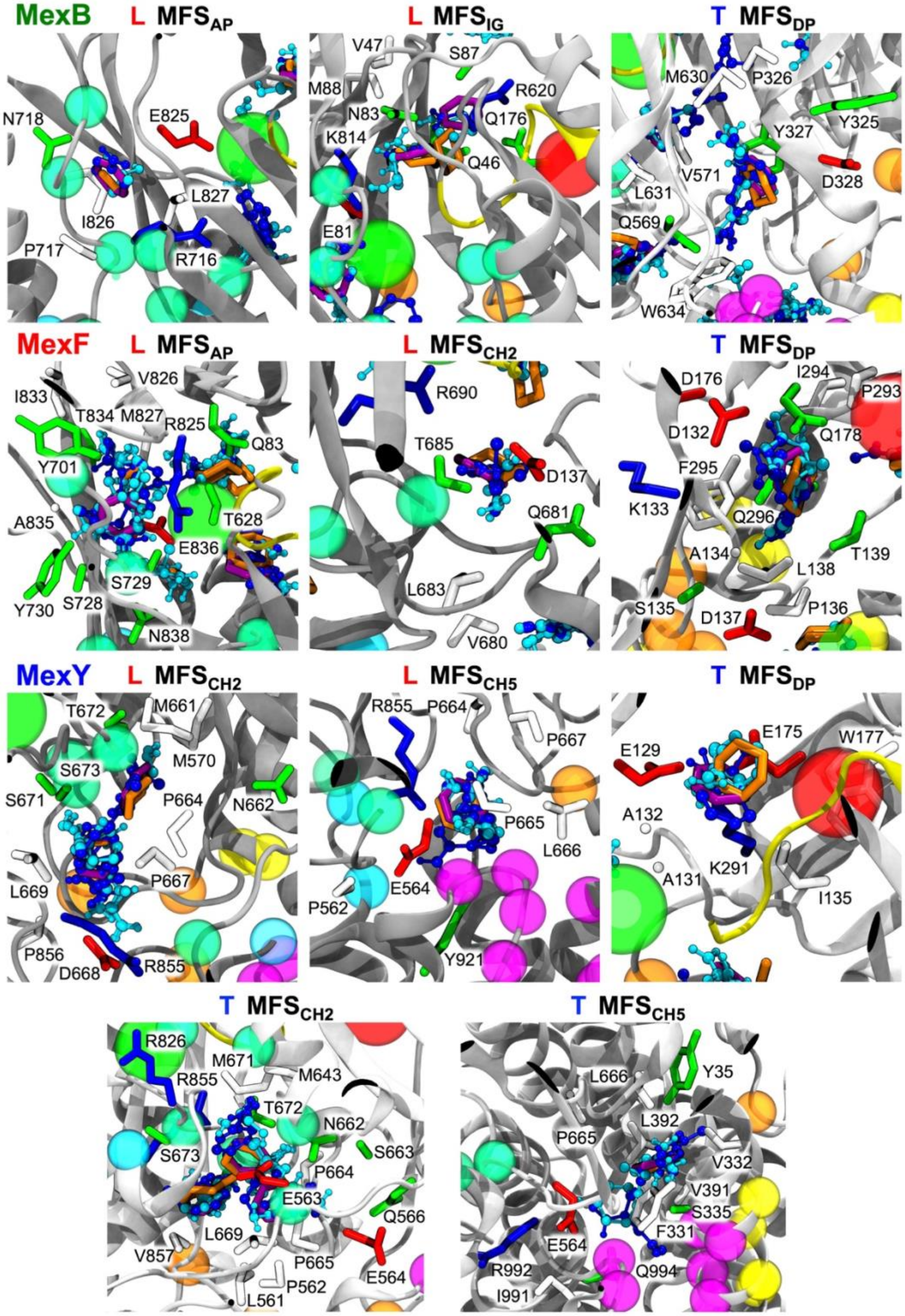


**Figure S9** Zoomed views of MFSs located in L and T protomers of MexB, MexF and MexY reported in Figure 4. Sidechains of protein residues interacting with each MFS, defined as those amino acids having at least one atom within 3.5 Å of any atom of the MFS small organic probes, are shown with the same color code of Figure 3. Polar, negatively charged, positively charged, and hydrophobic sidechains are colored in green, red, blue, and white, respectively. Due to overlapping MFSs and CSs, only MFSs of interest are shown in each MexY panel, other adjacent MFSs and CSs are omitted.

**Figure S10**

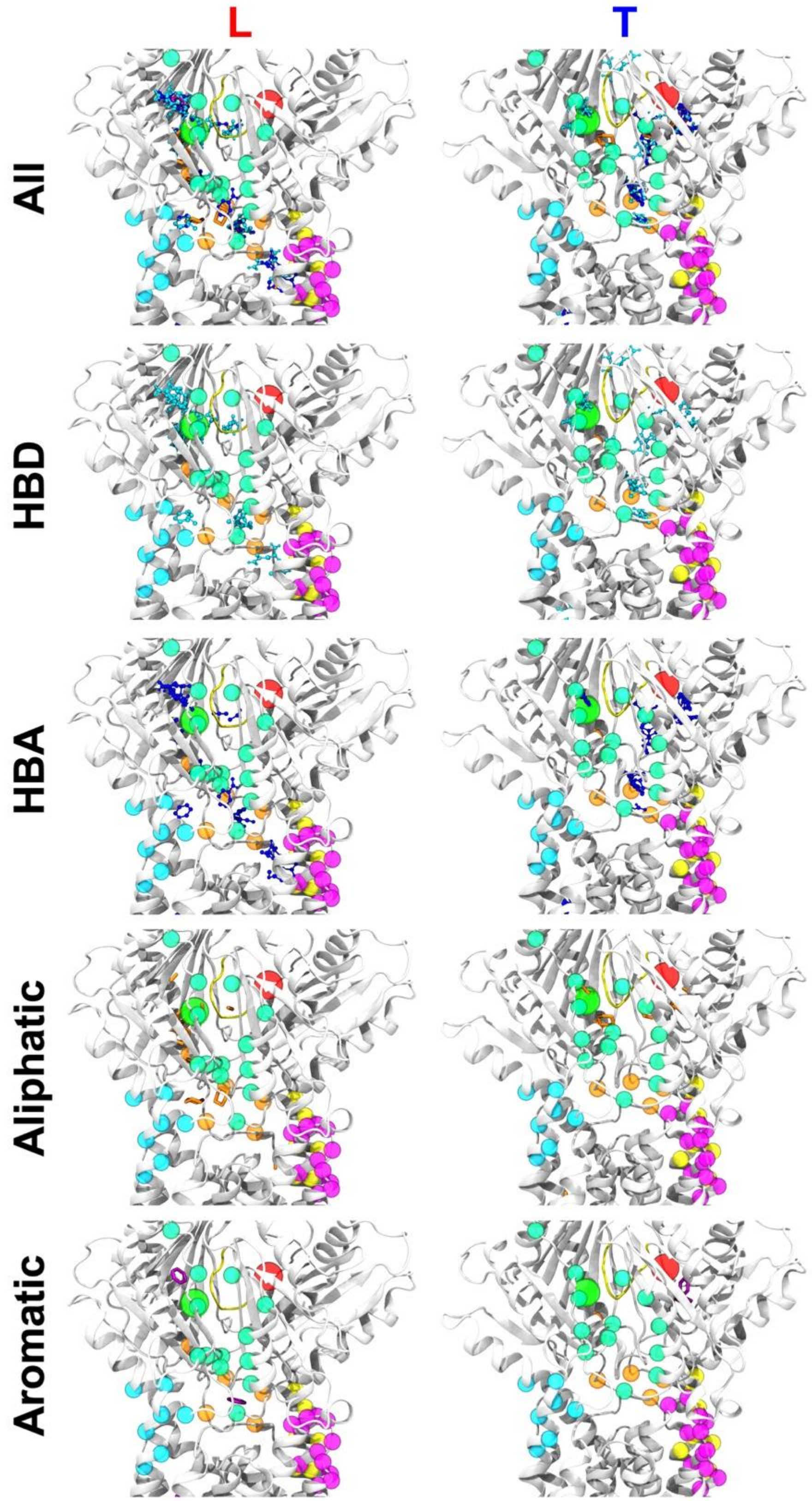


**Figure S10** Distributions of different types of organic probes bound to channel residues of MexF L and T protomers calculated on the most populated structure representative from MD simulations. The same color codes of Figures 1, 4, and 5 are applied to the protein and the small organic ligands, respectively.

**Figure S11**

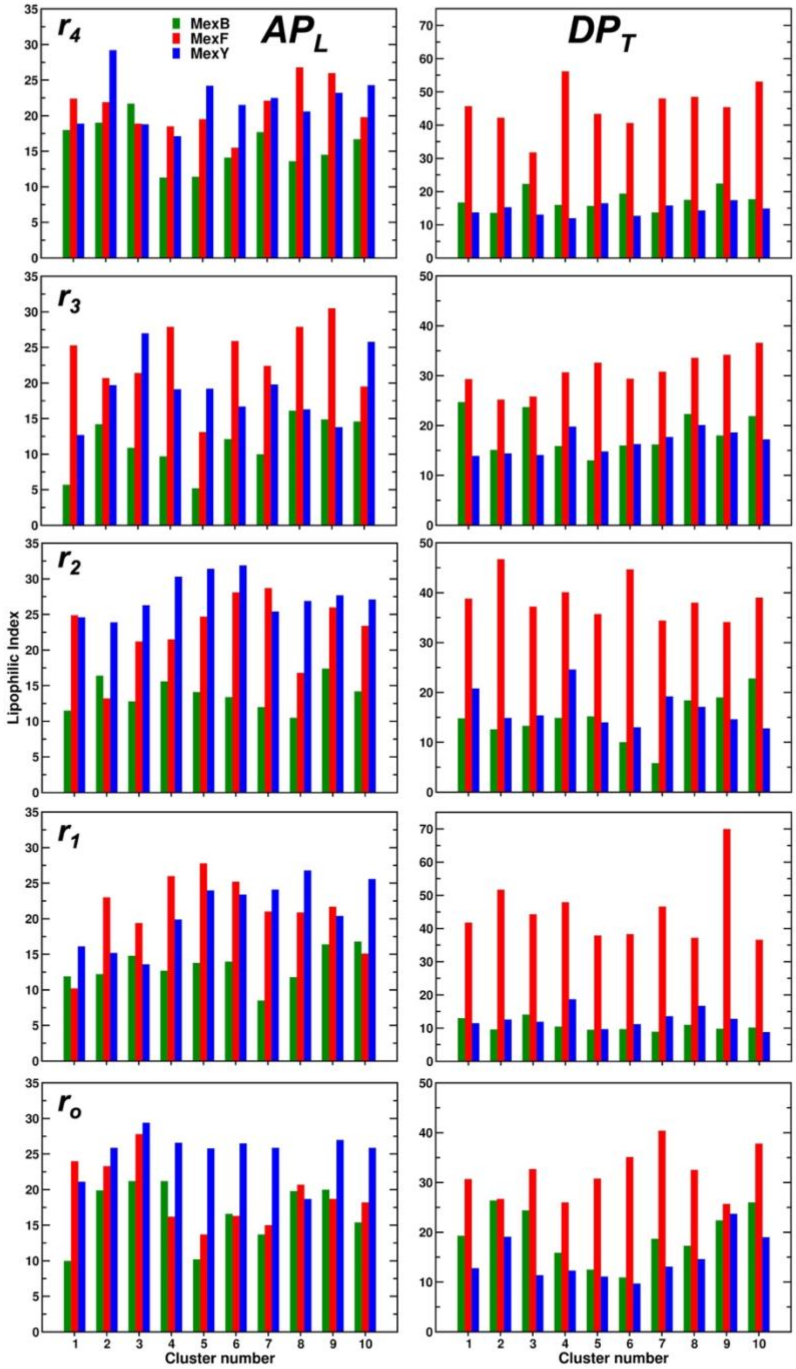


**Figure S11** Distribution of lipophilic indexes for $AP_L$ (left panel) and $DP_T$ (right panel) of MexB (green), MexF (red), and MexY (blue) over the cluster representatives extracted from replicas ($r_0$ - $r_4$) of equilibrated MD simulations of Mex RND transporters.

**Figure S12**

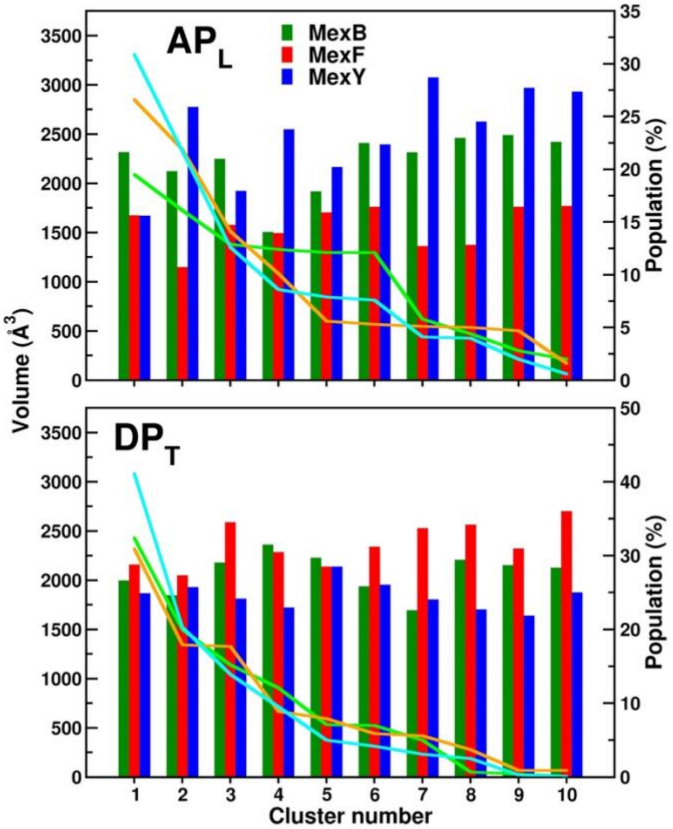


**Figure S12** Volumes of AP$_L$ and DP$_T$ binding pockets of MexB, MexF, and MexY top cluster structures extracted from the clustering analysis of MD simulations. Populations of each cluster of MexB, MexF, and MexY are shown with light green, orange, and cyan lines, respectively.

**Table S1** (Left) Structural alignment of MexF (colored domain-wise) and MexB (in white). For clarity, only a single protomer is shown. (Right) The results of each evaluation criteria for the MexF homology model obtained using the MexB X-ray crystal structure  (PDB ID 3W9I) as a template.

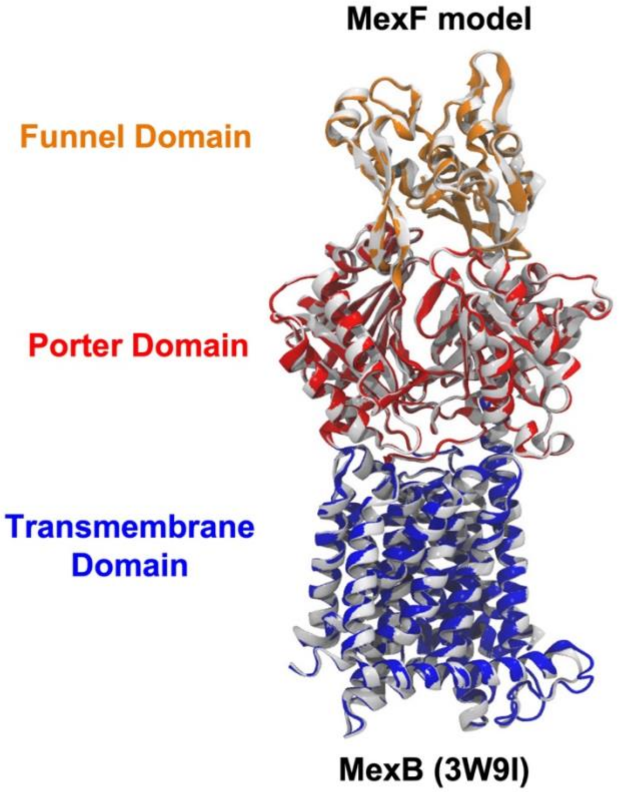


| Evaluation Criteria | MexF homology model |
|---|---|
| RMSD * (Cα-atoms) | 0.6 Å |
| TM-score | 0.99 |
| Ramachandran favored | 91.9% |
| Errat | 87.3% |
| Verify-3D | Pass |

*RMSD was calculated by performing the structural alignment with the *cealign* command of PyMOL (Schrödinger, 2015).

**Table S2** Amino acid residues of AP, DP and HP-trap regions of MexB and its isoforms.

| System | Protein binding domains | | |
|---|---|---|---|
| | **AP** | **DP** | **HP-trap** |
| **MexB** | I78 S79 S80 I90 T91 V92 V133 K134 N135 K292 L293 P565 D566 E567 L572 F573 A574 Q575 V576 Q577 T578 A618 G619 S623 S624 G625 A661 M662 V663 F664 A665 F666 A667 P668 P669 L672 E673 L674 G675 N676 A677 F680 D681 L682 V715 R716 P717 N718 G719 M824 E825 I826 L827 G828 | V45 Q46 V47 M88 T89 I90 I127 R128 V129 T130 K131 F136 L137 M138 V139 V140 F175 Q176 V177 F178 G179 S180 Q181 Q273 D274 Y275 S276 I277 N278 P326 Y327 D328 L572 F573 A574 V609 F610 T611 V612 T613 G614 F615 N616 F617 A618 G619 R620 G621 A627 F628 I629 | F136 F178 F573 F610 F615 F628 |
| **MexF** | M80 S81 S82 L92 T93 I94 S135 P136 D137 F295 Q296 P574 Q575 Q576 L581 V582 A583 F584 A585 Q586 L587 F627 T628 N632 S633 G634 A670 Y671 I672 A673 I674 F675 P676 P677 P678 Q681 G682 L683 G684 T685 I686 F689 R690 L691 V726 F727 S728 S729 Y730 A835 E836 L837 N838 G839 | V47 R48 A49 L90 T91 L92 I129 T130 V131 D132 K133 L138 T139 M140 V141 V142 V177 Q178 L179 F180 G181 L182 G183 D184 N276 Q277 Y278 A279 L280 R281 V329 Y330 D331 L581 V582 A583 A618 F619 P620 G621 L622 S623 I624 N625 G626 F627 T628 N629 S630 V636 F637 T638 | L138 F180 V582 F619 I624 F637 |
| **MexY** | T78 K79 A80 L89 T90 L91 A132 D133 S134 K291 M292 P562 E563 E564 F569 M570 I571 M572 V573 M574 Q575 Y613 G614 S618 S619 A620 R656 T657 V658 Y659 A660 M661 N662 S663 P664 P667 D668 L669 G670 S671 T672 F675 D676 F677 V710 M711 F712 A713 G714 V819 Q820 P821 R822 G823 | V45 S46 A47 A87 S88 L89 I126 Y127 V128 E129 K130 I135 Q136 L137 I138 V139 V174 E175 T176 W177 G178 A179 E180 S272 E273 Y274 G275 F276 V277 P325 Y326 D327 F569 M570 I571 A604 Y605 A606 V607 G608 G609 F610 S611 L612 Y613 G614 D615 G616 I622 F623 A624 | I135 W177 M570 Y605 F610 F623 |

**Table S3** Amino acid residues of CH1, CH2, CH3, CH4 and CH5 channels of MexB and its isoforms.

| System | Protein channels | | | | |
|---|---|---|---|---|---|
| | **CH1** | **CH2** | **CH3** | **CH4** | **CH5** |
| **MexB** | S835 G837 A839 A841 L867 G869 Q871 | D566 F645 K649 M653 F656 M662 N676 T678 R714 R716 N718 S721 E829 | N33 A37 P100 G296 N298 | I27 N298 L300 D301 S334 I337 H338 V341 | A335 S336 E339 V340 K342 T343 I989 S990 T991 G992 A993 G994 |
| **MexF** | S846 G848 A850 A852 L879 G881 T883 | Q575 G654 A658 A662 A665 Y671 T685 G687 P723 F727 S729 V732 A840 | S35 E39 L102 G299 N301 | L29 N301 I303 E304 R337 I340 E341 V344 | G338 S339 A342 V343 H345 T346 I1001 S1002 T1003 G1004 A1005 G1006 |
| **MexY** | Q830 R832 A834 Q836 V863 G865 Q867 | E563 G640 E644 Q648 A651 T657 S671 S673 N709 M711 A713 G716 P824 | A33 D37 A99 G295 N297 | I27 N297 V299 A300 E333 I336 R337 V340 | I334 S335 K338 V339 S341 T342 L985 S986 S987 G988 A989 G990 |

**Table S4** Sequence identity and similarity between RND transporters of *E. coli* and *P. aeruginosa*. Sequence similarities are shown in parentheses. All values are in percentages.

| System | MexF | MexY | AcrB | AcrD |
|---|---|---|---|---|
| **MexB** | 41.2 (59.4) | 46.9 (65.5) | 69.8 (83.2) | 61.1 (76.5) |
| **MexF** | - | 39.0 (57.7) | 39.7 (60.5) | 40.0 (59.5) |
| **MexY** | | - | 47.9 (67.0) | 48.2 (66.7) |
| **AcrB** | | | - | 65.3 (79.7) |

**Table S5** Chemical composition of AP and DP of MexB and its isoforms. All percentages were estimated using the sequence alignment of **Figure S2** performed with Clustal Omega.

| System | AP | | | DP | | |
|---|---|---|---|---|---|---|
| | **Charged** | **HP** | **Polar** | **Charged** | **HP** | **Polar** |
| **MexB** | 15<br>6 (+)<br>9 (–) | 55 | 30 | 10<br>6 (+)<br>4 (–) | 55 | 35 |
| **MexF** | 6<br>4 (+)<br>2 (–) | 51 | 43 | 12<br>6 (+)<br>6 (–) | 53 | 35 |
| **MexY** | 16<br>7 (+)<br>9 (–) | 52 | 32 | 14<br>2 (+)<br>12 (–) | 53 | 33 |

(+/–): percentage of positively/negatively charged residues. HP: hydrophobic

**Table S6.** Normalized weighted averages of different types of small organic probes identified in the proximity of relevant sites in MexB, MexF, and MexY transporters, calculated along the cumulative equilibrium MD trajectory. a) Probes identified at the $AP_L$ and $DP_T$ binding sites; b) Probes at the entry gates of CH1 to CH5 and at the IG in both the L and T protomers. Averages were calculated over the 5 most populated conformational clusters of each site and normalized by the number of different probes within each type (see **Figure S1**). Standard deviations, not reported for the sake of clarity, range from 0.1 to 0.3. The color code is the same used in Figure 1 to represent graphically the protein binding pockets (AP: green, DP: red), the G-loop (here dark yellow to differentiate it from CH4), and entry channels (CH1: cyan, CH2: seagreen, CH3: orange, CH4: yellow and CH5: magenta). The higher the number of probes the more intense the color.

a)

| PROBE | HBD | | HBA | | Aliphatic | | Aromatic | |
|---|---|---|---|---|---|---|---|---|
| **SITE** | **$AP_L$** | **$DP_T$** | **$AP_L$** | **$DP_T$** | **$AP_L$** | **$DP_T$** | **$AP_L$** | **$DP_T$** |
| **MexB** | 3.6 | 2.6 | 2.6 | 2.7 | 1.7 | 2.3 | 2.0 | 2.4 |
| **MexF** | 4.1 | 3.6 | 3.4 | 3.7 | 2.3 | 3.3 | 2.7 | 3.2 |
| **MexY** | 3.6 | 2.2 | 3.3 | 1.8 | 2.6 | 1.7 | 3.1 | 1.7 |

b)

| | PROBE | HBD | | HBA | | Aliphatic | | Aromatic | |
|---|---|---|---|---|---|---|---|---|---|
| | **MONOMER** | **L** | **T** | **L** | **T** | **L** | **T** | **L** | **T** |
| **CH1** | **MexB** | 0.0 | 0.0 | 0.0 | 0.0 | 0.0 | 0.0 | 0.0 | 0.0 |
| | **MexF** | 0.1 | 0.0 | 0.2 | 0.0 | 0.1 | 0.0 | 0.2 | 0.0 |
| | **MexY** | 0.0 | 0.1 | 0.1 | 0.1 | 0.1 | 0.0 | 0.1 | 0.1 |
| **CH2** | **MexB** | 1.5 | 2.1 | 1.2 | 1.8 | 0.7 | 1.1 | 1.0 | 1.4 |
| | **MexF** | 1.3 | 1.3 | 1.2 | 1.2 | 0.7 | 0.8 | 0.8 | 0.8 |
| | **MexY** | 1.7 | 2.1 | 1.7 | 2.0 | 1.2 | 1.2 | 1.4 | 1.6 |
| **CH3** | **MexB** | 0.2 | 0.1 | 0.2 | 0.1 | 0.1 | 0.1 | 0.3 | 0.1 |
| | **MexF** | 0.3 | 0.1 | 0.3 | 0.1 | 0.2 | 0.0 | 0.3 | 0.1 |
| | **MexY** | 0.3 | 0.2 | 0.5 | 0.3 | 0.4 | 0.2 | 0.5 | 0.2 |
| **CH4** | **MexB** | 0.1 | 0.0 | 0.1 | 0.1 | 0.2 | 0.2 | 0.3 | 0.2 |
| | **MexF** | 0.2 | 0.0 | 0.2 | 0.0 | 0.2 | 0.0 | 0.2 | 0.1 |
| | **MexY** | 0.2 | 0.2 | 0.3 | 0.2 | 0.3 | 0.2 | 0.4 | 0.3 |
| **CH5** | **MexB** | 0.2 | 0.4 | 0.2 | 0.4 | 0.1 | 0.3 | 0.3 | 0.5 |
| | **MexF** | 0.1 | 0.3 | 0.1 | 0.4 | 0.1 | 0.3 | 0.0 | 0.4 |
| | **MexY** | 0.8 | 0.6 | 0.9 | 0.6 | 0.6 | 0.4 | 0.7 | 0.5 |
| **IG** | **MexB** | 1.1 | 1.3 | 0.9 | 1.5 | 0.6 | 1.3 | 0.7 | 1.3 |
| | **MexF** | 1.9 | 1.6 | 1.7 | 1.6 | 1.3 | 1.5 | 1.3 | 1.2 |
| | **MexY** | 1.3 | 1.3 | 1.3 | 1.1 | 0.9 | 1.1 | 1.0 | 1.0 |

**Table S7** Lipophilic Indexes of $AP_L$ and $DP_T$ of MexB, MexF and MexY.

| System | Lipophilic Index | |
|---|---|---|
| | Pre-MD | MD clusters |
| **$AP_L$** | | |
| MexB | 10.5 | 19.9 ± 0.9 |
| MexF | 34.3 | 22.4 ± 3.1 |
| MexY | 12.1 | 25.8 ± 2.1 |
| **$DP_T$** | | |
| MexB | 27.8 | 19.0 ± 5.4 |
| MexF | 29.7 | 28.8 ± 2.7 |
| MexY | 15.4 | 20.7 ± 1.7 |

**Table S8** Volumes of $AP_L$ and $DP_T$ of MexB, MexF, and MexY.

| System | Volume (Å$^3$) | |
|---|---|---|
| | Pre-MD | MD clusters |
| **$AP_L$** | | |
| MexB | 1426 | 2152 ± 110 |
| MexF | 1480 | 1509 ± 85 |
| MexY | 1854 | 2242 ± 199 |
| **$DP_T$** | | |
| MexB | 2796 | 2038 ± 89 |
| MexF | 2588 | 2277 ± 94 |
| MexY | 3159 | 1868 ± 71 |
